# Synthetic Siglec-9 Agonists Inhibit Neutrophil Activation Associated with COVID-19

**DOI:** 10.1021/acscentsci.0c01669

**Published:** 2021-03-24

**Authors:** Corleone S. Delaveris, Aaron J. Wilk, Nicholas M. Riley, Jessica C. Stark, Samuel S. Yang, Angela J. Rogers, Thanmayi Ranganath, Kari C. Nadeau, The Stanford COVID-19 Biobank, Catherine A. Blish, Carolyn R. Bertozzi

**Affiliations:** † Department of Chemistry, Stanford University, Stanford, California 94305, United States; ‡ ChEM-H, Stanford University, Stanford, California 94305, United States; § Stanford Medical Scientist Training Program, Stanford University, Stanford, California 94305, United States; ∥ Stanford Immunology Program, Stanford University, Stanford, California 94305, United States; ⊥ Department of Medicine, Stanford University, Stanford, California 94305, United States; # Department of Emergency Medicine, Stanford University, Stanford, California 94305, United States; ∇ Sean N. Parker Center for Allergy and Asthma Research, Stanford University, Stanford, California 94305, United States; ◆ Stanford Biobank, Stanford University, Stanford, California 94305, United States; ○ Chan Zuckerberg Biohub, San Francisco, California 94158, United States; □ Howard Hughes Medical Institute, Stanford, California 94305, United States

## Abstract

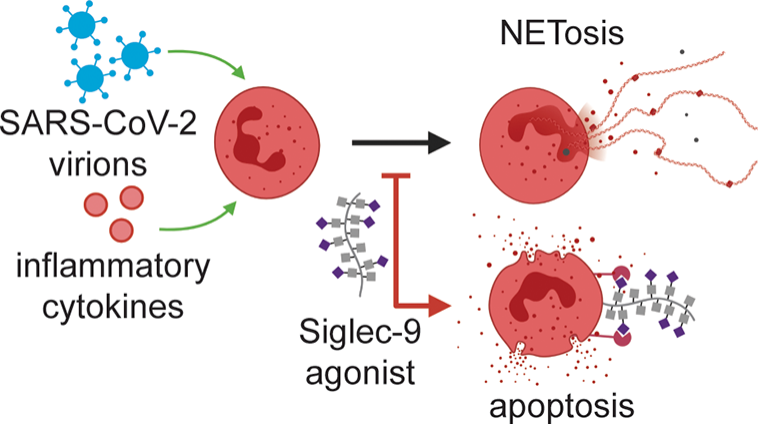

Severe cases of coronavirus disease 2019 (COVID-19), caused by infection with SARS-CoV-2, are characterized by a hyperinflammatory immune response that leads to numerous complications. Production of proinflammatory neutrophil extracellular traps (NETs) has been suggested to be a key factor in inducing a hyperinflammatory signaling cascade, allegedly causing both pulmonary tissue damage and peripheral inflammation. Accordingly, therapeutic blockage of neutrophil activation and NETosis, the cell death pathway accompanying NET formation, could limit respiratory damage and death from severe COVID-19. Here, we demonstrate that synthetic glycopolymers that activate signaling of the neutrophil checkpoint receptor Siglec-9 suppress NETosis induced by agonists of viral toll-like receptors (TLRs) and plasma from patients with severe COVID-19. Thus, Siglec-9 agonism is a promising therapeutic strategy to curb neutrophilic hyperinflammation in COVID-19.

## Abstract 2

In COVID-19, viral pathogen associated molecular patterns and viral-induced cytokines can induce NETosis of neutrophils at the site of infection and in the periphery. Siglec-9 agonists inhibit COVID-19 plasma-induced NETosis, potentially preventing deleterious hyperinflammatory responses.

## Introduction

**Figure 1:**
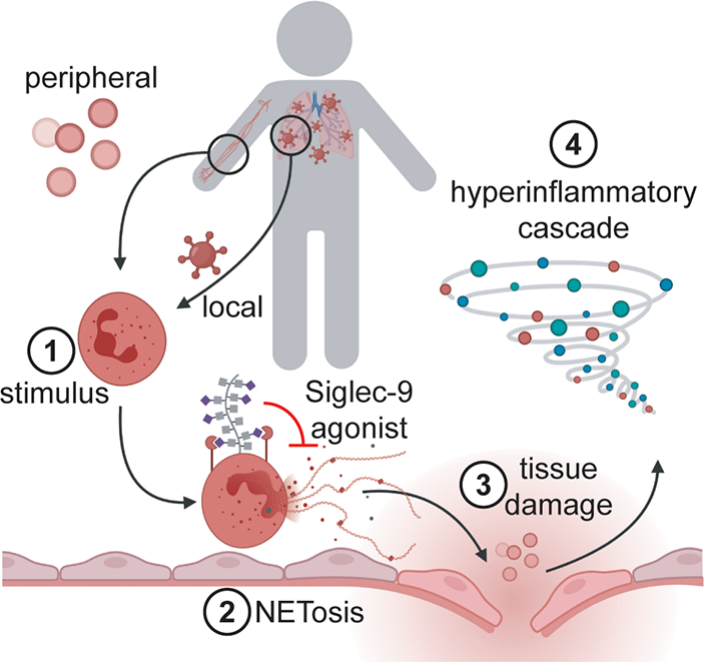
Local and peripheral inflammatory stimuli induce NETosis and a subsequent hyperinflammatory cascade in COVID-19. Both local inflammatory stimuli at the site of SARS-CoV-2 infection (e.g., virions) and peripheral inflammatory stimuli (e.g., the proinflammatory cytokines IL-8 and G-CSF) associated with COVID-19 have been shown to induce NETosis *in vitro*. These factors are suspected to be causative agents of NETosis *in vivo* as well, initiating a deleterious hyperinflammatory cascade leading to the symptoms of moderate and severe COVID-19. Agonists of the neutrophil-associated checkpoint receptor Siglec-9 could inhibit NETosis in COVID-19.

Runaway inflammation in coronavirus disease 2019 (COVID-19) is thought to lead to numerous complications, including potentially fatal pneumonia and acute respiratory distress syndrome (ARDS).^1−3^ While the specific causal factors of inflammation in COVID-19-related ARDS are unknown and likely multifarious, an emerging hypothesis posits that hyperactivation of neutrophils initiates and drives this response (Figure 1).^4−12^ Neutrophils are immune cells of the myeloid lineage that are involved in numerous innate immune functions. It has been suggested that neutrophils drive a hyperinflammatory response in COVID-19 through a death process called NETosis, in which neutrophils rapidly decondense chromatin and spew out a neutrophil extracellular trap (NET), an amalgam of genomic DNA, intracellular proteins (e.g., histones), and tissue-damaging enzymes (e.g., neutrophil elastase, myeloperoxidase).^13,14^ Extracellular DNA and tissue damage from NET-associated enzymes act as proinflammatory signals to other immune cells^15−17^ and are proposed to initiate the hyperinflammatory cascade in COVID-19, leading to ARDS and potentially death. Consistent with this hypothesis, NETs have been extensively observed both at the site of infection (i.e., pulmonary tissue)^18−21^ and in the periphery (i.e., sera and plasma).^19,21^

Both SARS-CoV-2 virions and serum/plasma from COVID-19 patients have been shown to induce NETosis of neutrophils isolated from healthy donors *in vitro*, consistent with the local and peripheral inflammatory responses observed in COVID-19.^19,21,22^ However, the specific signals that induce NETosis in viral disease remain an open question; viral ligands for toll-like receptors (TLRs), host damage-associated molecular patterns, antiviral cytokines (e.g., IL-8 and IFNγ), and activated platelets have all been implicated, but which if any of these is sufficient to induce NETosis is still debated.^21,23^ Beyond viral disease, NETosis has been linked to numerous inflammatory pathologies, including thrombosis and sepsis, both of which are observed in patients with COVID-19.^4^ During NETosis, inflammatory stimuli signal neutrophils to import calcium ions, which activate protein arginine deiminase 4 (PADI4).^24,25^ PADI4 mediates the conversion of arginine to the deiminated citrulline on histones.^25^ The loss of positive charges induces rapid unwinding of genomic DNA, which eventually ruptures the nucleus and the cell.^25^ When this happens, intracellular contents including genomic DNA, active PADI4, tissue-damaging NET-associated enzymes, and citrullinated histones are emitted into the extracellular space, all of which provoke an inflammatory response.^24,25^ Thus, strategies to curb neutrophil-mediated inflammation could treat both COVID-19 as well as other neutrophilic inflammatory pathologies.

Transcriptomic analyses of immune cells from severe COVID-19 patients show that neutrophils upregulate the myeloid checkpoint receptor Siglec-9, a member of the sialic acid-binding immunoglobulin-like lectin (Siglec) family that is also found on macrophages and activated T cells.^8,9,26−29^ This sialoglycan-binding immunosuppressive receptor has an intracellular signaling domain similar to the prominent lymphoid checkpoint receptor PD-1 and the myeloid suppressive receptor SIRPα.^30−32^ Clustering of Siglec-9 by ligand engagement leads to inhibitory signaling that quenches activation of the immune cells.^28^ Both erythrocytes and host-mimicking pathogens have been shown to engage Siglec-9 to suppress neutrophil-mediated immunity, including inhibiting NETosis.^28,33−35^ Furthermore, engagement of Siglec-9 on primary neutrophils has been shown to induce apoptotic pathways,^26^ in a manner similar to the engagement of Siglec-8 on eosinophils as occurs with an FDA-approved Siglec-8 agonist for eosinophilic inflammatory conditions.^36^ Given that Siglec-9 is both an anti-inflammatory and pro-apoptotic checkpoint molecule, we hypothesized that engagement of Siglec-9 could simultaneously inhibit proinflammatory NETotic cell death and induce quiet apoptotic cell death in COVID-19-related inflammation. Notably, an agonist of the related myeloid checkpoint receptor Siglec-10 (CD24Fc, trade name SACCOVID) has recently shown great promise in suppressing viral hyperinflammation and is in a Phase III clinical trial.^37,38^ However, unlike the case for Siglec-10 for which CD24 is a high-affinity and specific ligand, there is no comparable glycoprotein ligand known for Siglec-9 and thus no biological starting point for the design of therapeutic agonists.^39^

**Figure 2:**
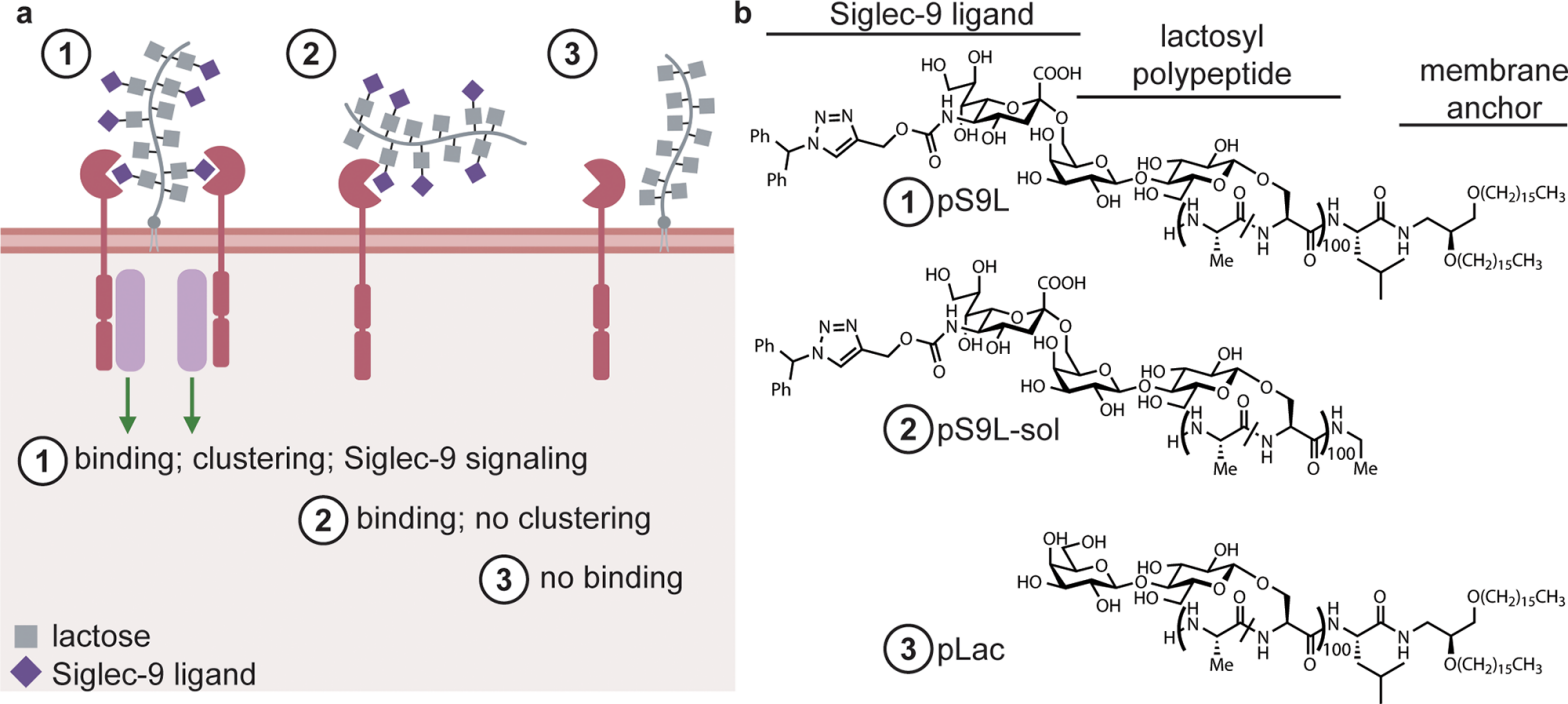
Synthetic glycopolypeptides bearing high-affinity Siglec-9 ligands engage Siglec-9 and induce clustering and signaling. (a) Membrane-anchored, cis binding glycopolypeptide **1** (pS9L) induces Siglec-9 signaling, while a soluble control polypeptide **2** (pS9L-sol) or a nonbinding but membrane-anchored control polypeptide **3** (pLac) does not. (b) Structures of the polypeptides **pS9L**, **pS9L-sol**, and **pLac**. Polypeptides are all based on an *O*-lactosyl poly serine-*co*-alanine scaffold and in some cases bear terminal Siglec-9-binding sialic acid analogues and/or C-terminal membrane-anchoring lipids.

We recently reported^40^ the design and synthesis of a potent Siglec-9 agonist comprising a lipid-conjugated glycopolypeptide bearing modified sialic acid residues that Paulson and co-workers had previously found to bind Siglec-9 with high affinity and specificity^41^ (pS9L, Figure 2). The lipid group enabled passive insertion into cell membranes, leading to engagement of Siglec-9 in cis on macrophage cell surfaces. This cell-surface clustering, in turn, induced Siglec-9 signaling and suppressed macrophage activation.^40^ We hypothesized that this was due to induced distribution of Siglec-9 into actively signaling clusters that we and others have observed.^28,40^ In this former study, we also designed control glycopolypeptides lacking either Siglec-9 binding glycans (i.e., the lactose-functionalized glycopolypeptide pLac) or a membrane anchoring lipid moiety (i.e., the soluble glycopolypeptide pS9L-sol) (Figure 2). Notably, potent Siglec-9 agonism required membrane anchoring and cis-engagement; the soluble analogue pS9L-sol was unable to stimulate Siglec-9 signaling and suppress macrophage activity, which we proposed was due to clustering efficiency of membrane-associated ligands binding cis versus soluble ligands binding in trans. We hypothesized that pS9L might also suppress neutrophil activation and NETosis by clustering Siglec-9 on neutrophils.

Here, we demonstrate that a synthetic cis-binding Siglec-9 agonist (pS9L, Figure 2b)^40^ inhibits NETosis in primary neutrophils in models of local (TLR-7/8 agonist) and peripheral (COVID-19 plasma) COVID-19-associated inflammation. Using live cell microscopy, we showed that TLR-7/8 activation by the nucleoside analogue resimiquod (R848) induces NETosis in primary human neutrophils. R848 treatment induces rapid citrullination of histone substrates, consistent with PADI4-mediated NETosis, and this process was blocked by Siglec-9 signaling induced by pS9L. Significantly, pS9L inhibited neutrophil NETosis induced by treatment with plasma from severe COVID-19 patients. In light of these data, we propose that Siglec-9 agonists could be therapeutic agents that inhibit COVID-19-associated inflammation.

## Results and Discussion

### TLR-7/8 Agonist R848 Induces NETosis of Primary Neutrophils *in Vitro*

In COVID-19, evidence of extensive NETosis can be observed in infected lungs,^18−21^ and SARS-CoV-2 virions have been shown to infect and induce NETosis of healthy neutrophils *in vitro*.^20^ These reports implicate TLR-7 and/or TLR-8 in inducing NETosis of neutrophils at the site of infection.^20,42^ Notably, TLR-7 and TLR-8 are single-stranded RNA receptors with numerous substrates identified in the SARS-CoV-2 genome.^43^ Consistent with the hypothesis that SARS-CoV-2 induces TLR-7/8-mediated immunity, human genetic variations in *TLR7* are associated with severe COVID-19.^44^ Thus, agonists of TLR-7/8 may provide a convenient means of modeling local inflammation induced by viral infection *in vitro* without using live virus.

We assayed TLR agonists using the live-cell imaging techniques described by Gupta and co-workers.^45^ In this assay, freshly isolated neutrophils are cultured in low-serum media in the presence of a fluorogenic and membrane impermeable DNA-intercalating dye (Cytotox Green). Upon genomic DNA-externalization by NETosis, dye intercalates and fluorescence increases. As previously demonstrated,^45^ because NETs are much larger than the nuclei of apoptotic cells, NETotic cells yield much larger areas of fluorescence than apoptotic cells, as observed by microscopy. Thus, apoptotic cells can be filtered out by only counting large (i.e., ≫100 μm^2^) fluorescent objects.

**Figure 3:**
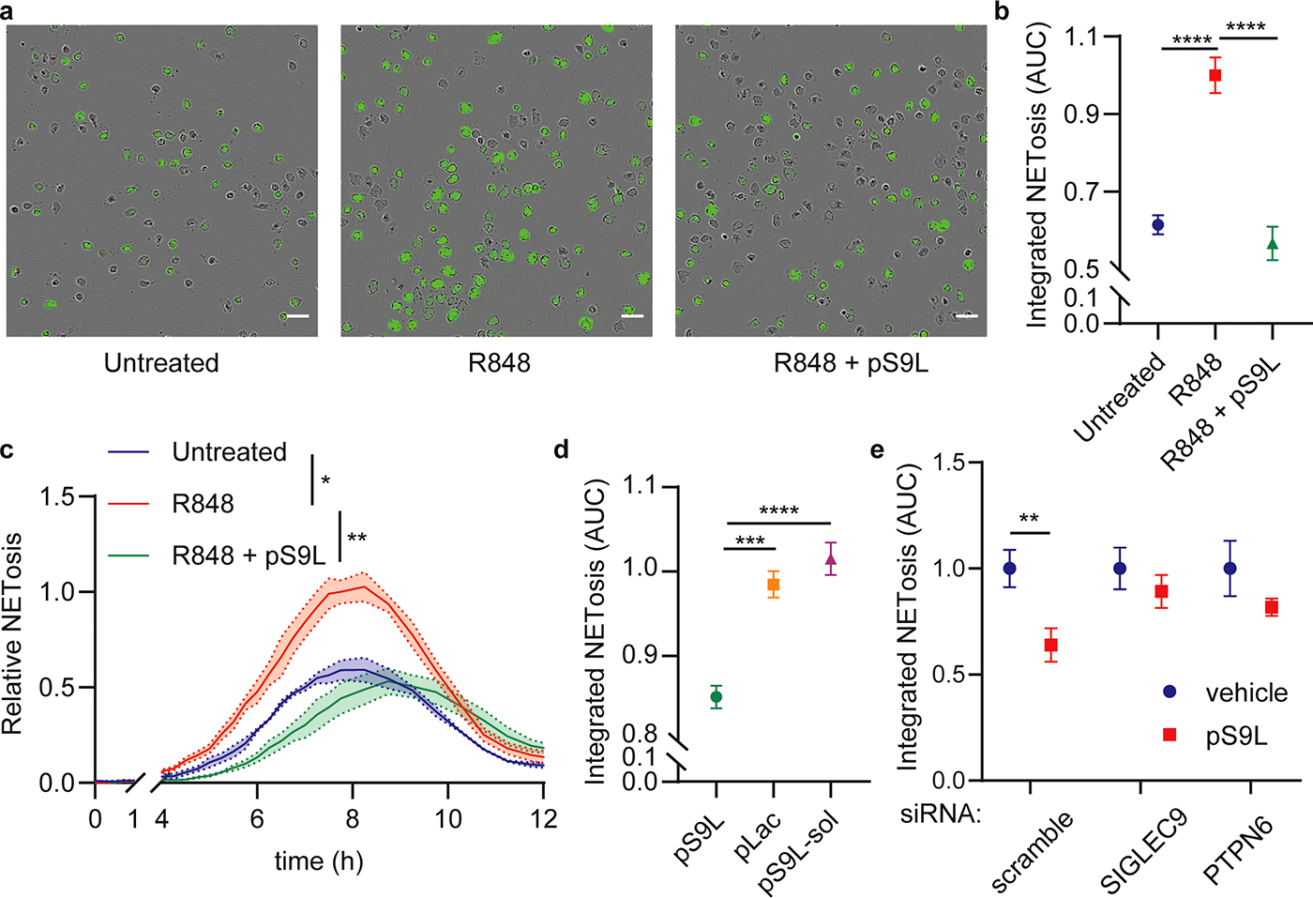
A cis-binding Siglec-9 agonist (**pS9L**) inhibits R848-induced NETosis via Siglec-9 and SHP-1. (a–c) Primary neutrophils were cotreated with R848 (10 μM) and glycopolypeptide (500 nM) in IMDM supplemented 0.5% hiFBS containing the membrane impermeable DNA intercalators Cytotox Green or Red (250 nM). Images were acquired by fluorescence microscopy every 15 min for 12 h. The area of all green fluorescent objects >300 μm^2^ was quantified, and the total area was averaged across three images per well. Relative NETosis was determined by normalizing to the maximal NET area from R848 treatment alone (*t* = 8 h). (a) Representative phase contrast and fluorescence images from *t* = 8 h. Scale bars indicate 40 μm. (b) Quantitation of NETosis over time as area under the curve in (c). Error bars represent SD. (c) NET formation and degradation as a function of time. Error bands represent SEM. (d) Treatment of R848-stimulated neutrophils with various glycopolypeptides. Error bars represent SD. (e) HL-60 cells were transfected with siRNAs against SIGLEC9 (encoding Siglec-9), PTPN6 (encoding SHP-1), or a scrambled control and then grown for 2 days. Cells were then cotreated with R848 (10 μM) and vehicle or pS9L (500 nM). Relative NETosis is determined as in (b), except all objects >200 μm^2^ were quantified and the R848 maximum in dHL-60s was observed at 2.5 h post induction. Error bars represent SD. Statistics were determined by two-way ANOVA (c) or one-way ANOVA (b,d,e). * *p* < 0.05; ** *p* < 0.01; *** *p* < 0.001; **** *p* < 0.0001.

We found that a TLR-7/8 agonist, R848, was sufficient to induce NETosis of healthy neutrophils *in vitro* (Figure 3a–c, Figure S1). We also assayed the citrullination status of the PADI4 substrate H3 by Western blot and observed that R848 rapidly induced citrullination at R2, R8, and R17 (Figure S2). While citrullination is an important aspect of NETosis,^24^ the extent of citrullination is not necessarily indicative of the extent of NETosis as, for example, PMA-induced NETosis only yields moderate citrullination (Figure S2).^46^ Additionally, we performed quantitative phosphoproteomics^47^ with lysates of neutrophils treated with media, phorbol-12-myristate-13-acetate (PMA), or R848 (Figure S3, Table S1). We observed results similar to previously published data sets using neutrophils stimulated with either R848^48^ or PMA.^49^ Furthermore, several phosphosites were found to be differentially regulated in both data sets, including those involved in neutrophil degranulation and calcium flux, consistent with the described mechanism of NETotic cell death.^24,49^ These results indicate that the TLR-7/8 agonist R848 induces NETosis in primary neutrophils. Thus, this compound can be used to model local inflammation associated with viral infection, including in COVID-19.

### A Siglec-9 Agonist Inhibits TLR-7/8-Induced NETosis via SHP-1

Previous work by von Gunten, Varki, and their respective co-workers has shown that engagement of Siglec-9 leads to apoptotic and nonapoptotic death pathways as well as immunosuppression in neutrophils.^26,50^ Thus, we hypothesized that Siglec-9-mediated immunosuppression and cell death could override the NETotic effect of antiviral TLR signaling. To test this notion, we used our previously described Siglec-9 agonist, **pS9L**(40) as well as the two control glycopolypeptides **pLac** and **pS9L-sol** (Figures 2 and S4). We assayed anti-NETotic activity by cotreatment of glycopolypeptide (500 nM) with R848 (10 μM) in primary neutrophils in the live-cell assay described above (Figure 3). We observed that **pS9L** was sufficient to inhibit NETosis induced by R848 treatment (Figure 3a–c). Moreover, neither control polymer inhibited R848-induced NETosis (Figure 3d). We also confirmed that **pS9L** inhibits NETosis comparably to high concentrations of cross-linked anti-Siglec-9 antibody (clone 191240) (Figure S5).^51,52^ Previously, von Gunten and co-workers described the generation of mitochondrial-derived reactive oxygen species (ROS) as an important signaling step of Siglec-9-induced apoptotic signaling.^26^ We found that treatment with pS9L, in the absence of any TLR ligand so as to avoid NADPH-derived ROS in inflammatory signaling, induced an oxidative burst, as did treatment with a cross-linked anti-Siglec-9 antibody (Figure S6). Furthermore, the oxidative burst was inhibited by the addition of the SHP-1/2 inhibitor NSC-87877, suggesting that SHP-1 and/or SHP-2 mediate **pS9L**-induced oxidative burst in neutrophils, consistent with Siglec-9 engagement (Figure S6b).

We performed quantitative phosphoproteomics using lysates of R848-stimulated primary neutrophils cotreated with vehicle, **pS9L**, or **pLac** (Figure S3, Table S2). Notably, we found increased phosphorylation of hyccin (HYCCI/FAM126A), a key component in phosphorylation of phosphoinositides,^53^ a class of signaling molecules implicated in mediating NETosis.^54^ Additionally, we observed increased phosphorylation of RASAL3 (RASL3), a negative regulator of the MAPK signaling pathway.^55^ These data suggest that **pS9L** inhibits the calcium flux and NADPH activity necessary for NETosis, as well as the MAPK-suppressive effects that have been previously described for **pS9L** in macrophages.^40^

To determine whether the anti-NETotic effect of **pS9L** is specifically mediated by Siglec-9 signaling, we recapitulated our results in the promyelocytic leukemia cell line HL-60. These cells can be differentiated into neutrophil-like cells (dHL-60) using all-trans retinoic acid (ATRA, 100 nM) and dimethyl sulfoxide (DMSO, 1.25% v/v). Notably, dHL-60 cells have previously been used to study NETosis *in vitro*.^31,56^ Consistent with those prior reports, R848 induced NETosis in dHL-60 cells (Figure S7). Furthermore, we observed that **pS9L** inhibited NETosis and that siRNA knockdown of Siglec-9 (encoded by *SIGLEC9*) or SHP-1 (encoded by *PTPN6*) abrogated the effect of **pS9L** (Figures 3e, S8 and S9). Therefore, the Siglec-9 agonist **pS9L** inhibits TLR7/8-induced NETosis via Siglec-9 and SHP-1.

### Siglec-9 is Upregulated in Severe COVID-19 and Can Suppress NETosis Induced by COVID-19 Plasma

**Figure 4:**
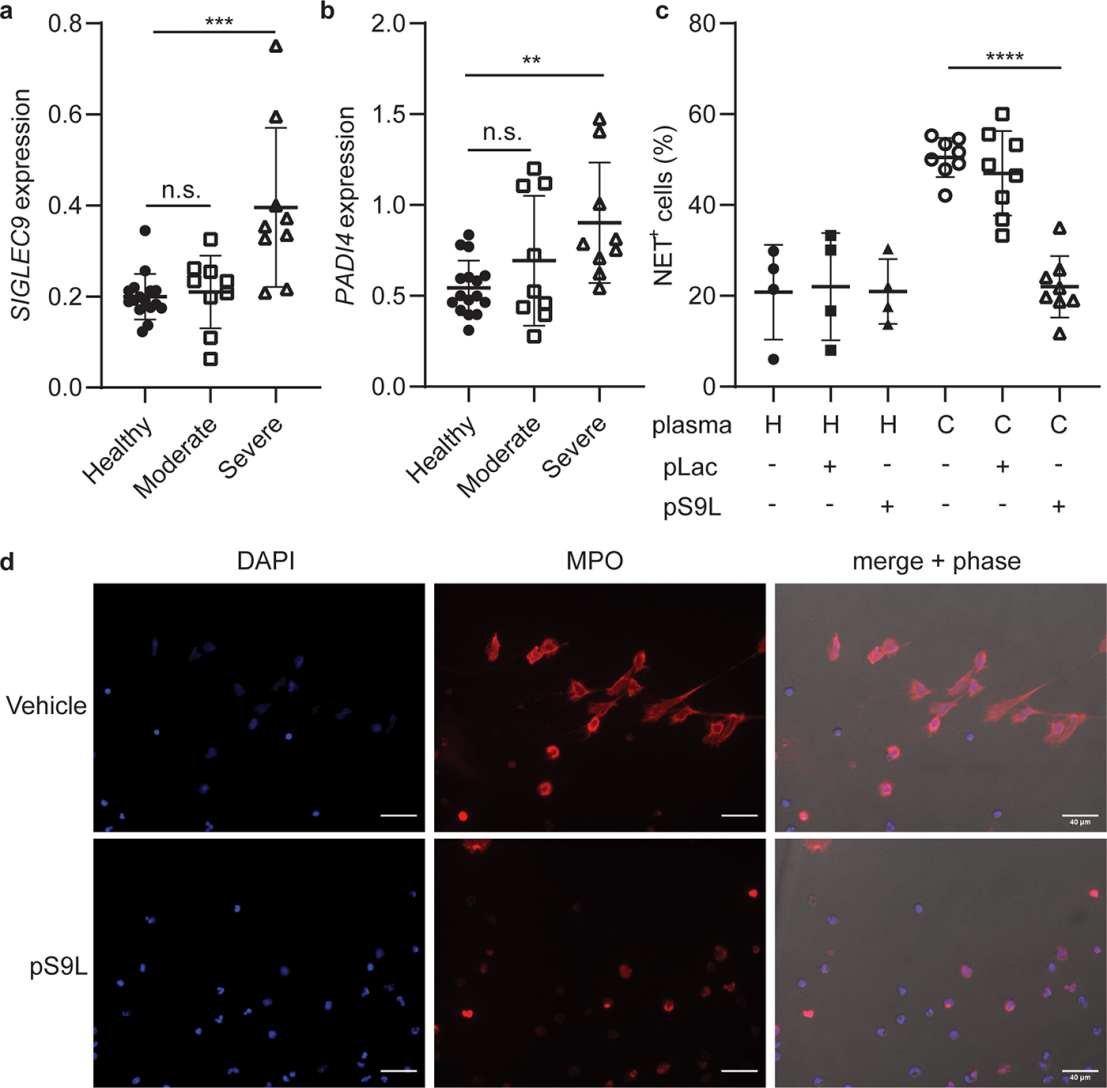
Siglec-9 agonist **pS9L** inhibits NETosis of neutrophils induced by COVID-19 plasma. (a, b) Analysis of publicly available single-cell transcriptomics data^8^ for *SIGLEC9* expression (a) and *PADI4* expression (b) on neutrophils in peripheral blood from healthy donors or COVID-19 patients. Error bars represent SD. Statistics were determined using mixed effects model. ** = *p* < 0.01; *** = *p* < 0.001 (c, d) Primary neutrophils were cultured in undiluted and citrate anticoagulated plasma from healthy donors or COVID-19 patients for 4 h. Cells were fixed, stained for extracellular myeloperoxidase, and imaged in DAPI imaging media by fluorescence microscopy. Cells were treated in technical triplicate and imaged across multiple fields of view. (c) Proportion of NET-positive cells (%) across all fields of view. Each dot represents and individual plasma sample. (d) Representative images from a COVID-19 patient plasma sample with or without pS9L. Error bars represent SD. Statistics were determined using mixed effects models to account for samples using repeat neutrophil donors. **** = *p* < 0.0001.

Sera and plasma from COVID-19 patients have been shown to induce NETosis of neutrophils isolated from healthy donors *in vitro*.^19,21^ The causative components are unclear; however, potential factors include viral TLR ligands, damage-associated molecular patterns that bind TLRs, activated platelets, and (pro)inflammatory cytokines. Recent reports have described increased levels of neutrophil-activating cytokines in COVID-19 plasma, predominantly IL-8 and G-CSF.^57^ We also observed that the combination of IL-8 and G-CSF was sufficient to induce NETosis *in vitro* (Figure S10). Additionally, transcriptomic analyses of peripheral myeloid cells^8^ and neutrophils^9^ in COVID-19 patients have revealed increased *SIGLEC9* expression (Figure 4a, S11) and *PADI4* expression (Figures 4b and S11). We hypothesize that this is an exhaustion-like phenotype in which Siglec-9 expression is induced on hyper-NETotic neutrophils, similar to what has been observed with Siglec-9 on exhausted tumor-infiltrating T cells.^51^ These observations further support Siglec-9 an attractive target for therapeutic blockade of hyperinflammatory NETosis in COVID-19.

To test the hypothesis that **pS9L** can inhibit NETosis induced by COVID-19 plasma, we treated neutrophils isolated from whole blood of healthy donors with citrate-anticoagulated heterologous plasma from healthy donors or COVID-19 patients. Neutrophils in undiluted plasma were cotreated with **pS9L** (500 nM), the nonbinding analogue **pLac** (500 nM), or vehicle. To satisfy biosafety restrictions, cells were incubated in the presence of COVID-19 plasma for 4 h and then fixed before assaying for extracellular complexes of myeloperoxidase (MPO) and DNA (DAPI) (Figure 4c,d). The combination of these stains, which when observed extracellularly is indicative of NETosis, has been previously used to identified NET^+^ cells in the context of COVID-19.^20^ We observed that COVID-19 plasma induced NETosis of neutrophils from healthy donors, as indicated by the formation of web-like NET structures (Figure 4d). As in previous experiments with R848, COVID-19 plasma-stimulated NETosis was inhibited by **pS9L** treatment (Figure 4c,d). Furthermore, **pLac** did not inhibit NETosis induced by COVID-19 plasma, and neither **pS9L** nor **pLac** affected basal NETosis of *in vitro* cultured neutrophils (Figure 4c). We performed similar experiments staining neutrophils treated with 10% plasma in IMDM (Figure S12) or undiluted plasma (Figure S13) for extracellular H1/DNA complexes, another marker of NETs,^58−60^ and observed comparable results.

Collectively, these data demonstrate that Siglec-9 agonism inhibits NETosis induced by COVID-19 patient plasma and thus could inhibit peripheral inflammation in patients with COVID-19. Additionally, Siglec-9 agonists could resolve NET-associated pathologies observed in COVID-19 and elsewhere such as immunothrombosis^21^ and sepsis.^4,5^

### Safety Statement

For experiments using plasma from patients with COVID-19, all experiments were performed in a certified BSL-2+ biosafety cabinet with appropriate institutional approval for working with blood products derived from patients with COVID-19. All items that came in contact with plasma were disinfected with 10% bleach for 30 min or fixed in 4% formaldehyde solution for 15 min before being removed from the biosafety cabinet. Otherwise, no unexpected or unusually high safety hazards were encountered.

## Conclusion

We have demonstrated that Siglec-9 agonists can inhibit NETosis induced by COVID-19-associated proinflammatory signals. Thus, Siglec-9 is a therapeutic target to inhibit potentially fatal hyperinflammation associated with COVID-19 in an analogous fashion to the highly effective therapeutics currently aimed at the Siglec-10/CD24 interaction. A CD24-Fc fusion has been shown to engage Siglec-10 as an immune checkpoint on macrophages and sequester the nuclear protein HMGB1, which can act as a damage associated molecular pattern by engaging TLR4.^61^ The Siglec-9 agonists described here have previously been shown to inhibit macrophage TLR4 signaling and engage macrophage Siglec-9.^40^ Thus, Siglec-9 agonists may be multipurpose therapeutics, able to inhibit both the clinically unaddressed problem of proinflammatory NETosis and also subsequent inflammatory signaling from tissue damage that is currently being clinically investigated.

The glycopolypeptides described here may have direct translational potential. As lipid conjugates, they may have sufficient reversible albumin binding activity as to achieve long plasma residence times approaching those of antibodies or Fc fusion proteins. As they are products of chemical synthesis, modifications to enhance preferred drug properties would be quite straightforward. Indeed, glycopolymers with lipid variants that enhance membrane association or plasma membrane residence time have been used for other purposes in our lab.^62,63^ More broadly, however, the work herein provides motivation to develop Siglec-9 agonists of any molecular classes, including monoclonal antibodies or Fc fusion proteins. Finally, Siglec-9 agonists have the potential to expand beyond ARDS to other NET-related pathologies such as thrombosis,^64,65^ atherosclerosis,^66^ and cystic fibrosis.^67^

## Supporting Information Available

Supplementary Figures S1–13; extended materials and methods; table of reagents (PDF)
Supplementary Tables S1 and S2, listing results of phosphoproteomics (XLSX)
