# Supplementary material for: Synthetic Siglec-9 Agonists Inhibit Neutrophil Activation Associated with COVID-19
Source: ACS Cent Sci. 2021 Mar 24;7(4):650–7. doi: 10.1021/acscentsci.0c01669 (PMC8009098; doi:10.1021/acscentsci.0c01669)
Supplement: Supplementary file 1 — oc0c01669_si_001.pdf [file oc0c01669_si_001.pdf]

Supporting Information

Synthetic Siglec-9 agonists inhibit neutrophil activation associated with COVID-19

Authors.

Corleone S. Delaveris,[1,2] Aaron J. Wilk,[3,4,5] Nicholas M. Riley,[1] Jessica C. Stark,[1] Samuel S. Yang,[6] Angela J. Rogers,[5] Thanmayi Ranganath,[5] Kari C. Nadeau,[5,7] the Stanford COVID-19 Biobank,[8] Catherine A. Blish,[5,9] Carolyn R. Bertozzi[1,2,10]

Author Affiliations.

[1]Department of Chemistry, Stanford University, Stanford CA, 94305

[2]ChEM-H, Stanford University, Stanford, CA 94305

[3]Stanford Medical Scientist Training Program, Stanford, CA 94305

[4]Stanford Immunology Program, Stanford University, Stanford, CA 94305

[5]Department of Medicine, Stanford University, Stanford, CA 94305

[6]Department of Emergency Medicine, Stanford University, Stanford, CA 94305

[7]Sean N. Parker Center for Allergy and Asthma Research, Stanford, CA, 94305

[8]Stanford Biobank, Stanford University, Stanford, CA 94305

[9]Chan Zuckerberg Biohub, San Francisco, CA 94158

[10]Howard Hughes Medical Institute, Stanford, CA 94305

Contents.

Supplementary Figures S1-S13 p. 2-18

Extended Materials and Methods p. 19-31

Supplementary Figures.

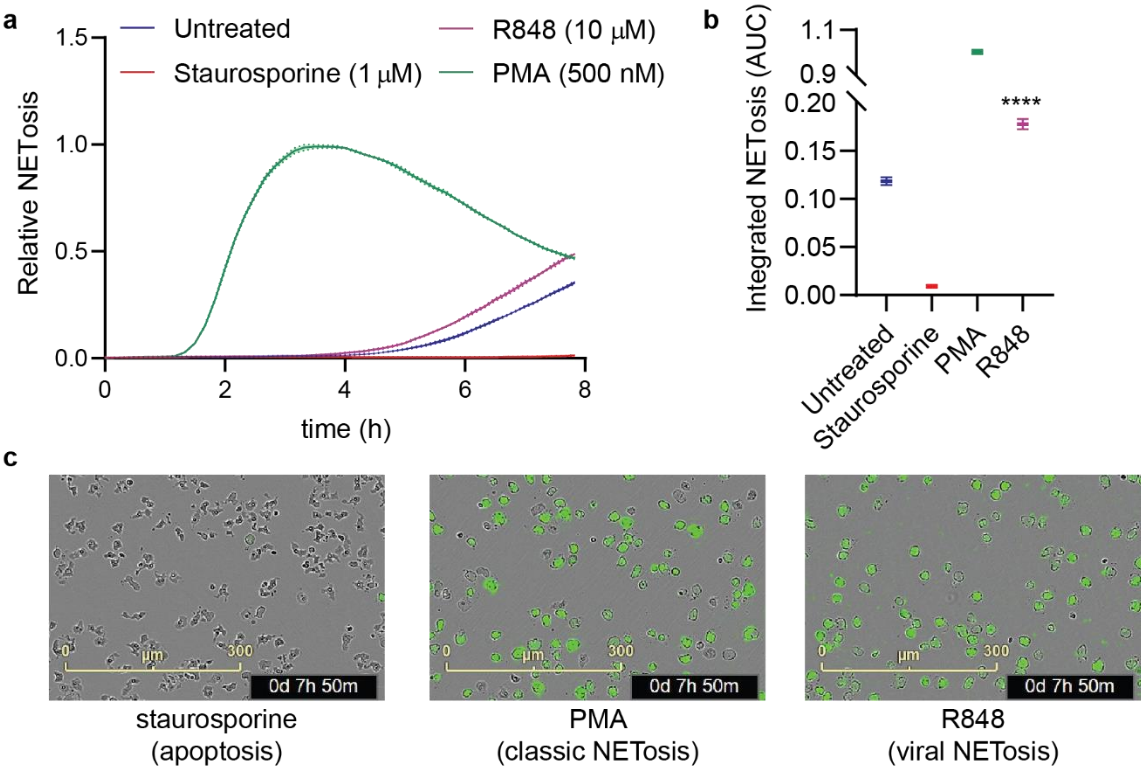

Figure S1.TLR-7/8 agonist R848 induces NETosis in primary neutrophils. (**a-c**) Primary neutrophils were treated with compounds at the concentrations detailed in (**a**) in IMDM supplemented 0.5% hiFBS containing the membrane impermeable DNA intercalator Cytotox Green (250 nM). Images were acquired by fluorescence microscopy every 10 min for 8 h. The area of all green fluorescent objects >300 μm^2 was quantified and averaged across three images per well. Relative NETosis was determined by normalizing to the maximal NET area from PMA treatment (t = 3 h). (**a**) Time-course data for NET formation and degradation over time. Error bands represent SEM. (**b**) Quantitation of (**a**) as area under the curve. Error bars represent SD. All data are representative of multiple independent experiments using neutrophils from different donors. **** p < 0.0001. (**c**) Representative images of neutrophils treated with staurosporine, PMA, or R848 stained with Cytotox Green at t = 7h 50m.

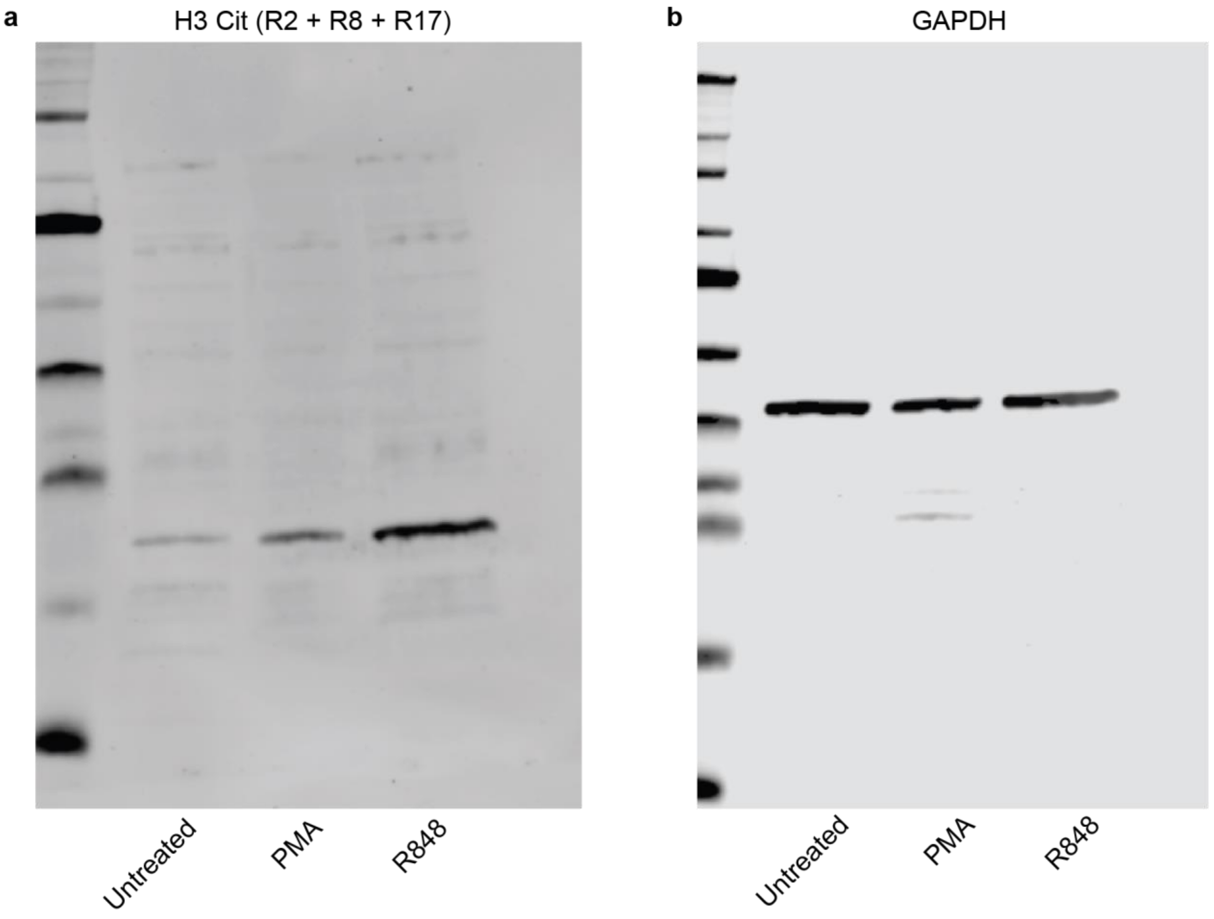

Figure S2. R848 induces rapid citrullination of H3 in primary neutrophils. Primary neutrophils were treated with compound in IMDM [+] 0.5% hiFBS for 30 min before being lysed and analyzed by Western blot for citrullination of histone H3. (**a**) H3Cit (R2 + R8 + R17). (**b**) GAPDH.

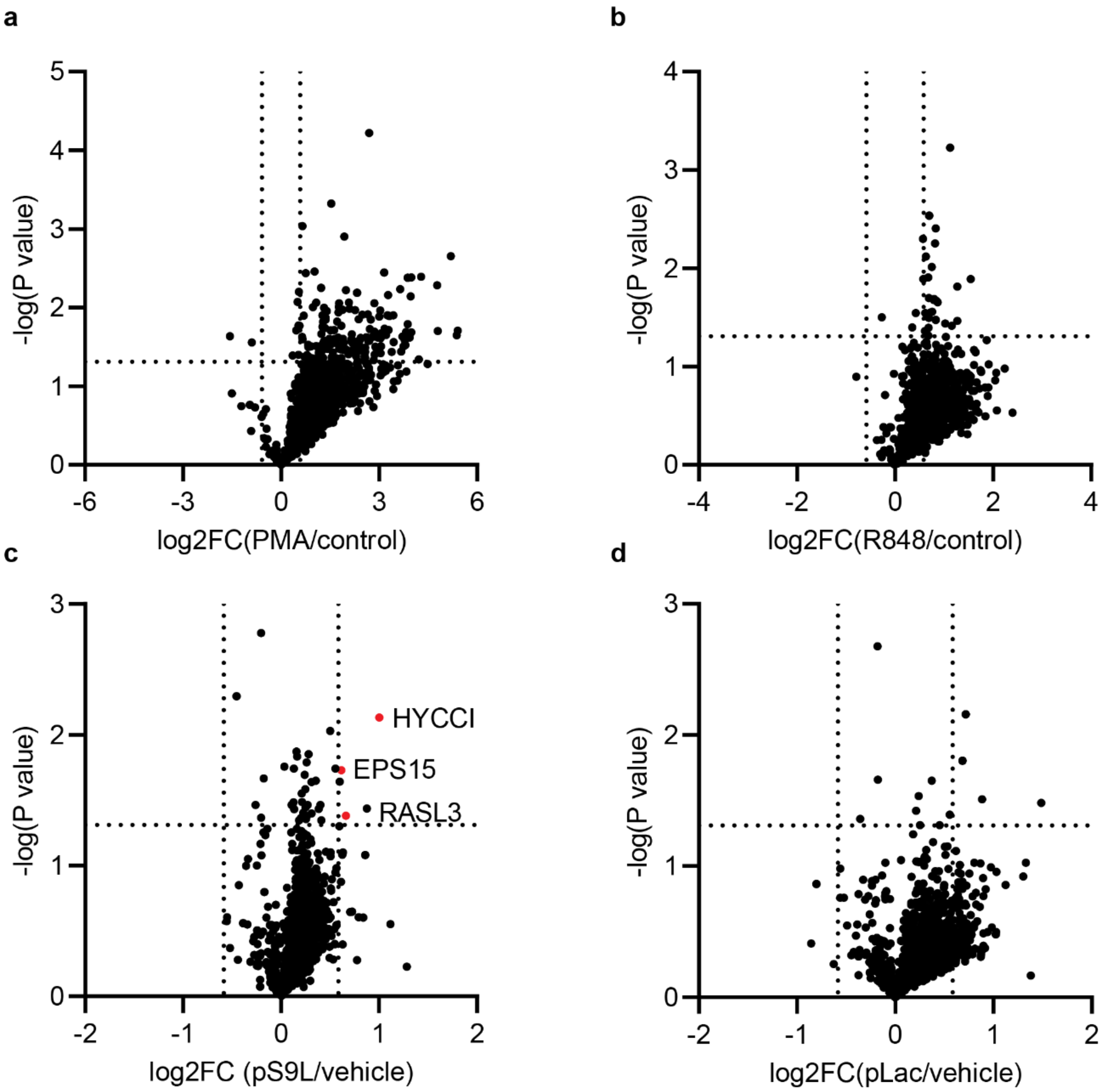

Figure S3. Volcano plots of significance vs. fold-change for phosphopeptides enriched from neutrophil lysates. Neutrophils isolated from healthy donor whole blood were treated with either PMA (500 nM) or R848 (10 µM) or left untreated for 30 min (**a**,**b**) or with R848 (10 µM) and one of pS9L (500 nM), pLac (500 nM) or vehicle for 15 min (**c**,**d**) in IMDM [+] 0.5% hiFBS. The cells were lysed, digested, and phosphopeptides were enriched before being TMT-labeled and analyzed by LC-MS. Vertical lines represent x = +/- 0.585 (corresponding to 1.5-fold change) and horizontal lines represent p = 0.05 as determined by a paired t-test to account for variability between neutrophil donors. Each datapoint represents the average fold-change from three

different donors. (**a**) Fold-change of PMA compared to untreated neutrophils. (**b**) Fold-change of R848 compared to untreated neutrophils. (**c**) Fold-change of pS9L-treated compared to vehicle-treated R848-stimualted neutrophils. Red dots highlight significant hits unique to this dataset compared to (**d**). (**d**) Fold-change of pLac-treated compared to vehicle-treated R848-stimualted neutrophils.

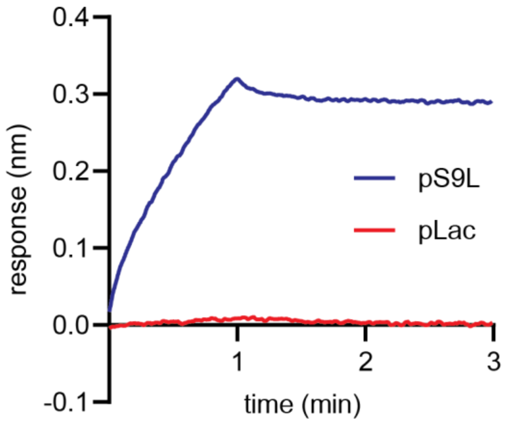

Figure S4. Siglec-9 binds pS9L but not pLac. Siglec-9-Fc was immobilized onto anti-hFc-coated tips on an OctetRed96 to a threshold of 0.4 nm. Association of glycopolypeptide (2.5 μM) was measured by dipping Siglec-9-Fc coated tips into a solution of glycopolypeptide in PBS with 0.1% BSA to abrogate nonspecific binding. Tips were conditioned prior to the first assay and regenerated between runs with three washes in 100 mM glycine buffer (pH 1.5).

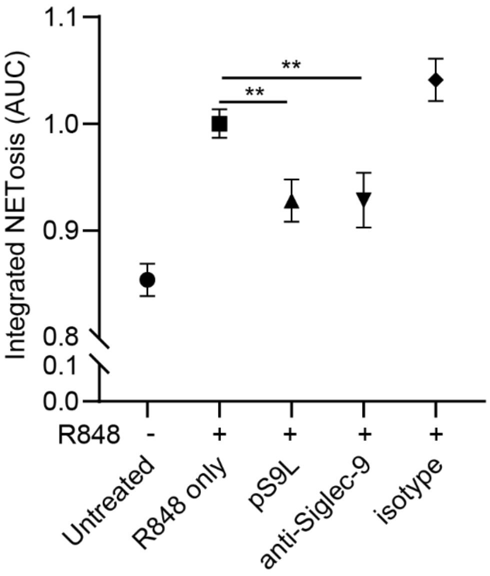

Figure S5. pS9L inhibits NETosis comparably to Siglec-9 agonist antibody. Primary neutrophils were cotreated with R848 (10 µM) and either cis-binding Siglec-9 agonist pS9L (500 nM) or antibody (anti-Siglec-9 clone 191240 or an isotype control IgG) (35 µg/mL) precomplexed with Protein A (5 µg/mL) or vehicle in IMDM supplemented with 0.5% hiFBS and containing Cytotox Green (250 nM). Images were acquired by fluorescence microscopy on an Incucyte Zoom every 1 h for 12 h. The area of all green fluorescent objects >200 µm^2 was quantified and averaged across three images per well. The area under the curve was quantified and normalized to the mean value for R848 treated neutrophils with no cotreatment.

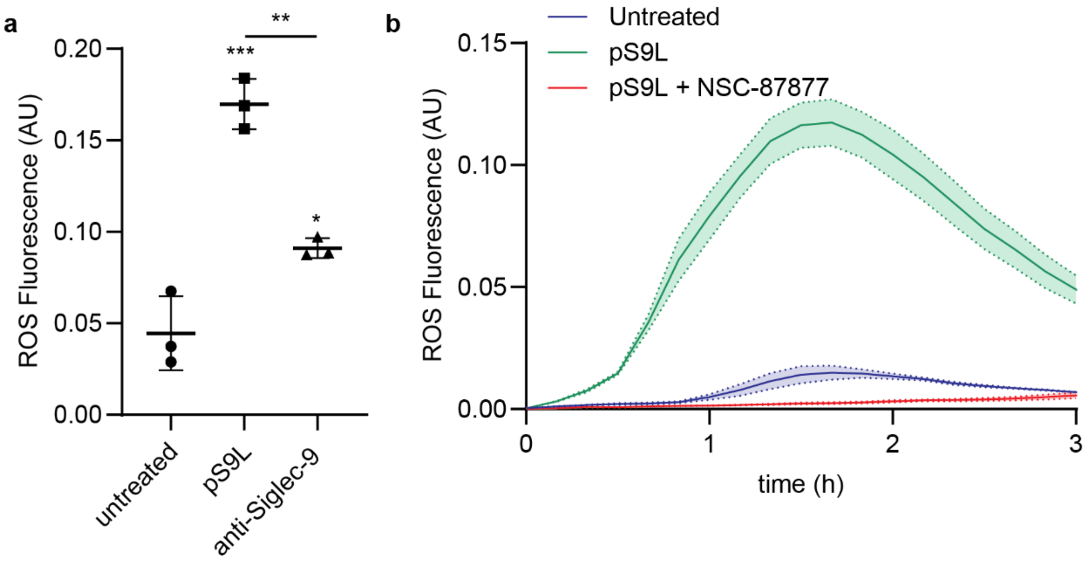

Figure S6. Siglec-9 engagement induces a SHP-1/2 dependent oxidative burst. (**a**) Primary neutrophils were treated with either cis-binding Siglec-9 agonist pS9L (500 nM) or anti-Siglec-9 (clone 191240) (35 µg/mL) precomplexed with Protein A (5 µg/mL) or vehicle in IMDM supplemented with 0.5% hiFBS and containing CellROX Deep Red (5 µM). Phase and red fluorescence images were acquired every 10 min using an Incucyte S3 in a 37 °C and 5% CO_2 incubator. ROS fluorescence was quantitated using integrated intensity normalized to confluence. Statistics were determined by one-way ANOVA. * = $p < 0.05$; ** = $p < 0.01$; *** = $p < 0.001$. (**b**) As in (**a**), with or without the SHP-1/2 inhibitor NSC-87877 (50 µM), as has been previously used to study SHP-1/2-mediated Siglec activity.[65]

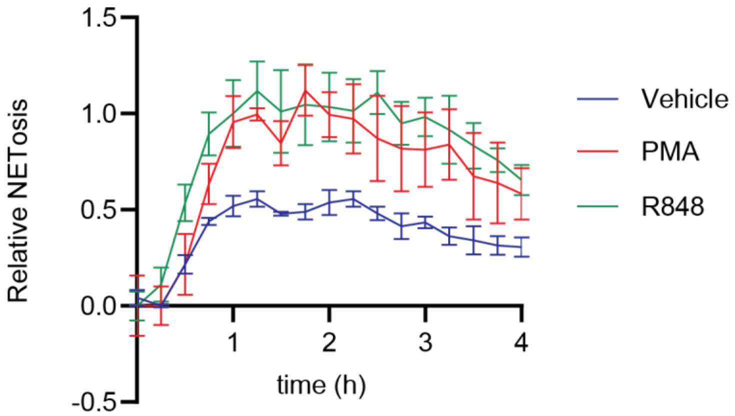

Figure S7. TLR-7/8 agonist R848 induces NETosis in dHL-60 cells. HL-60 cells were cultured in RPMI-1640 supplemented with 20% hiFBS in the presence of DMSO (1.25% v/v) and ATRA (100 nM) for 6 d. Cells were then seeded in serum-free RPMI-1640 containing Cytotox Green (250 nM) and NETosis was induced with PMA (100 nM), R848 (10 µM), or vehicle. Phase and green fluorescence images were acquired every 15 min using an Incucyte S3. The area of all green fluorescent objects >200 µm^2 was quantified and averaged across three images per well. Relative NETosis was determined by normalizing to the maximal NET area from PMA treatment alone (t = 2.5 h).

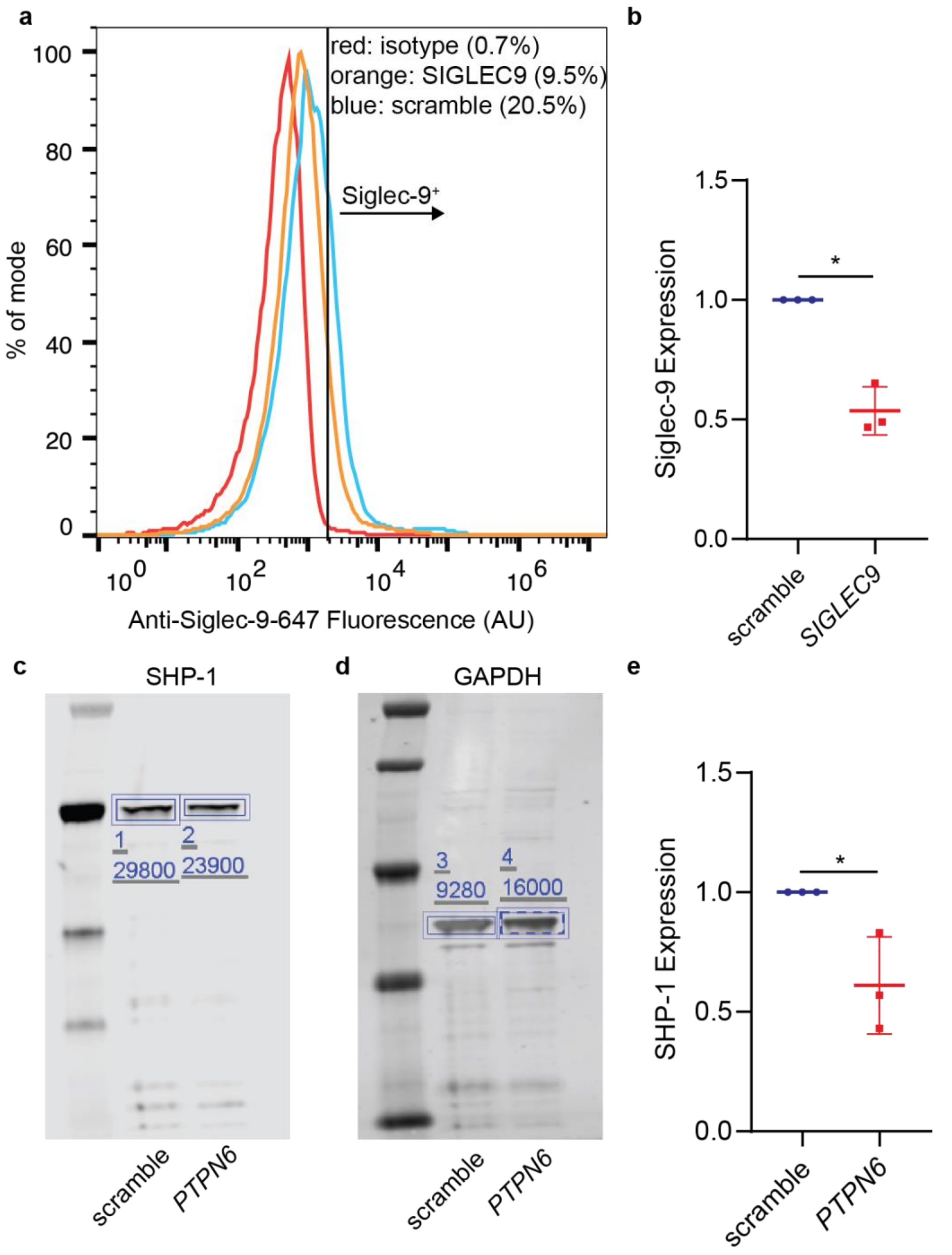

Figure S8. Siglec-9 and SHP-1 levels are reduced by siRNA knock-down of differentiated HL-60 neutrophil-like cells. HL-60 cells were cultured in RPMI-1640 supplemented with 20% hiFBS in the presence of DMSO (1.25% v/v) and ATRA (100 nM). After 4 d, the media was changed and cells were treated with siRNA's. On day 6, expression of target proteins was assayed. (**a**,**b**) dHL-60's treated with either SIGLEC9-targeting or scramble siRNA's were stained with an anti-

Siglec-9 antibody (clone K8, AlexaFluor647 conjugate) or an isotype control and analyzed by flow cytometry. (**a**) Representative histograms of dHL-60 cells treated with scramble or *SIGLEC9* targeting siRNAs. Gates were set such that < 1% of isotype-treated cells were Siglec-9^+. The gate used in this data set is shown and the percentages of cells Sigle-9^+ is shown in the legend in the upper right. (**b**) Siglec-9 expression was quantified percent of the population Siglec-9^+ (i.e., FL4-A^+) and normalized to the population treated with scramble siRNA across each of three different differentiations and transfections. Error bars represent SD. Statistics were determined by a paired t-test. * = $p < 0.05$. (**c**-**e**) Lysates from dHL-60's treated with siRNA's targeting *PTPN6* (encoding SHP-1) or a scrambled negative control were analyzed by Western blot, staining for either SHP-1 (**c**) or GAPDH (**d**). Fluorescence signal was quantitated by LiCOR. (**e**) SHP-1 expression was quantified by LiCOR and normalized to GAPDH fluorescence intensity in the same sample. GAPDH-normalized values were then normalized to the population treated with scramble siRNA across each of three different differentiations and transfections. Error bars represent SD. Statistics were determined by paired t-test. * = $p < 0.05$.

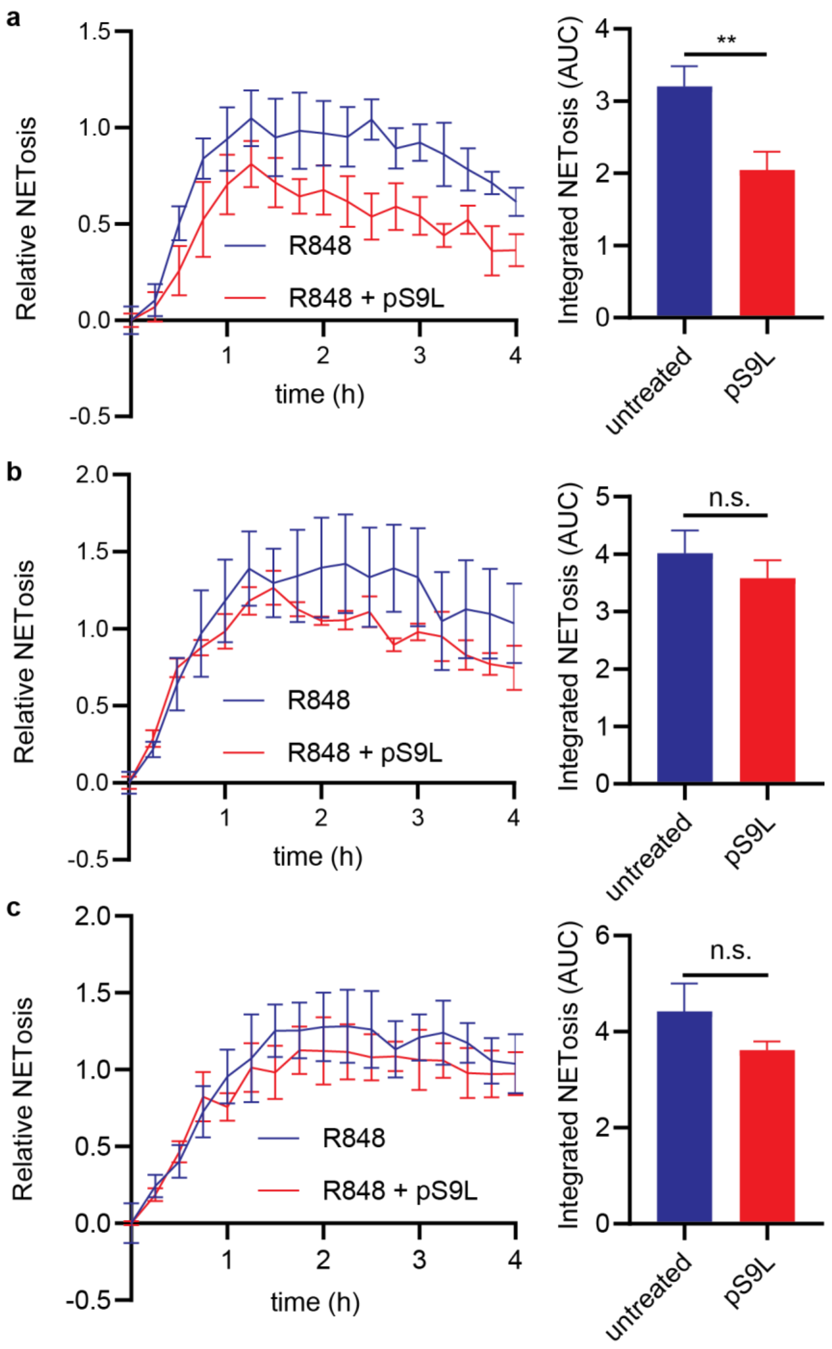

Figure S9. Cis Siglec-9 agonist pS9L inhibits NETosis via Siglec-9 and SHP-1. HL-60 cells were cultured in RPMI-1640 supplemented with 20% hiFBS in the presence of DMSO (1.25% v/v) and ATRA (100 nM). After 4 d, the media was changed and cells were treated with siRNA's. On day 6, cells were then seeded in serum-free RPMI-1640 containing Cytotox Green (250 nM) and NETosis was induced with R848 (10 µM) with or without pS9L (500 nM). Phase and green

fluorescence images were acquired every 15 min using an Incucyte S3. The area of all green fluorescent objects >200 μm^2 was quantified and averaged across three images per well. Relative NETosis was determined by normalizing to the maximal NET area from PMA treatment alone (t = 2.5 h). Area under the curve (AUC) was quantified and statistics were calculated using a two-way t-test. (**a**) Scramble siRNA control. (**b**) SIGLEC9 targeting siRNA cocktail. (**c**) PTPN6 siRNA. Error bars represent SD. ** = $p < 0.01$; n.s. = not significant.

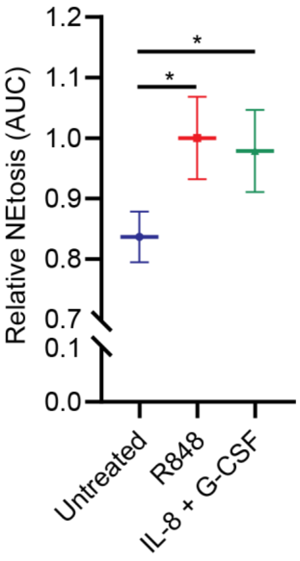

Figure S10. A combination of IL-8 and G-CSF induce NETosis in primary neutrophils. Primary neutrophils were stimulated with R848 (10 µM), a combination of IL-8 (100 ng/mL) and G-CSF (100 ng/mL), or vehicle and cultured in IMDM supplemented 0.5% hiFBS containing the membrane impermeable DNA intercalator Cytotox Green (250 nM). Images were acquired by fluorescence microscopy every 1 h for 8 h. The area of all green fluorescent objects >300 μm^2 was quantified and averaged across three images per well. Data were quantitated as area under the curve measurements. Error bars represent SD. Data are representative of multiple independent experiments using neutrophils from different donors. * $p < 0.05$.

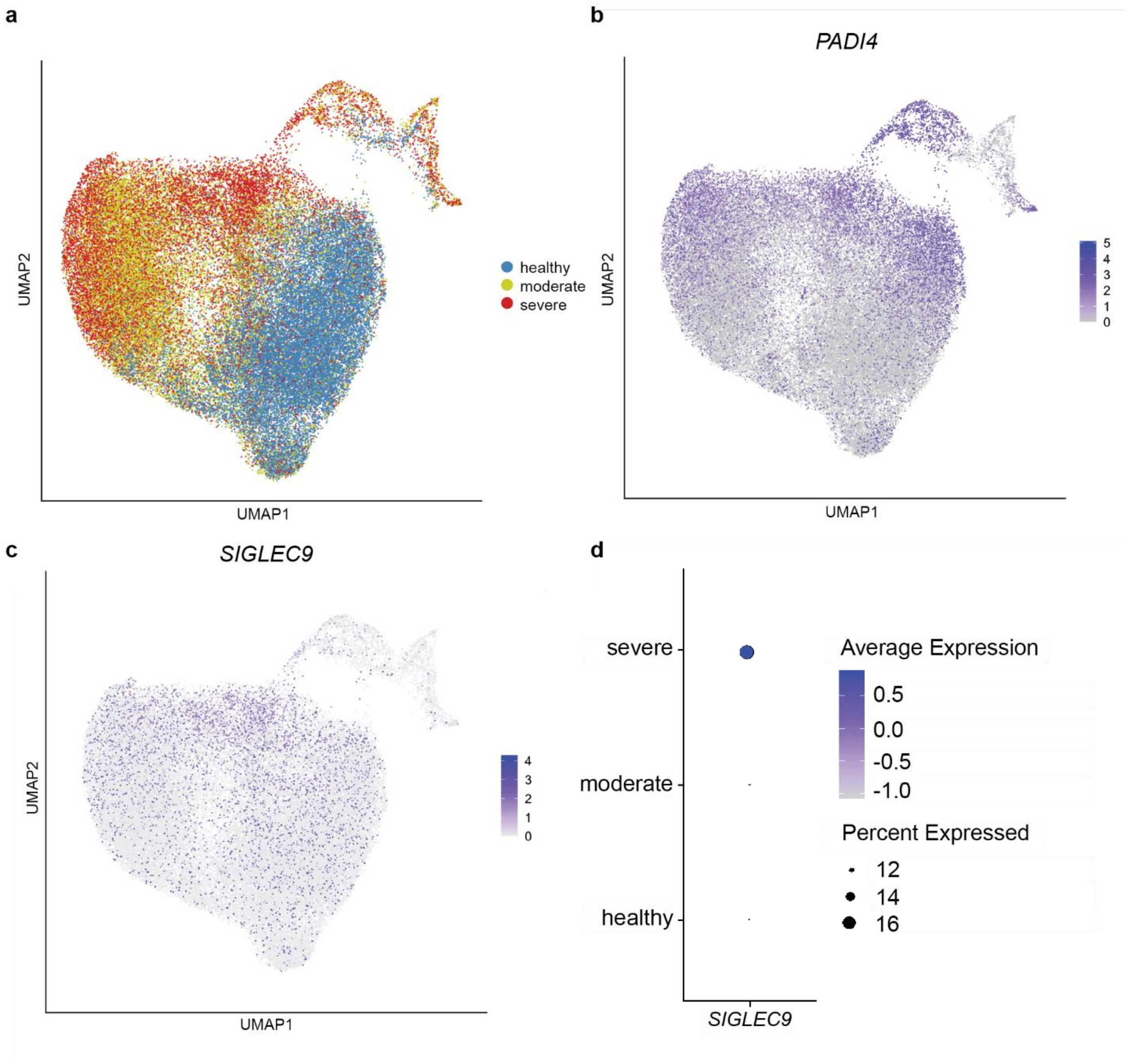

Figure S11. *SIGLEC9* and *PADI4* are upregulated by neutrophils of patients with severe COVID-19. (**a-c**) Uniform Manifold Approximation and Projection (UMAP) plot of neutrophils from the single-cell transcriptomic dataset published by Schulte-Schrepping and coworkers.[8] (**a**) Each cell is colored by the WHO severity score class (moderate, WHO score 4-5; severe, WHO score 6-8) of the patient at the time of sample collection, demonstrating strong severity-associated phenotypic reconfiguration of neutrophil transcriptome in COVID-19. (**b**) Each cell is colored by relative *PADI4* expression. (**c**) Each cell is colored by relative *SIGLEC9* expression.

(**d**) Dot plot depicting average and percent *SIGLEC9* expression by neutrophils in each WHO severity score class, indicating upregulation of *SIGLEC9* in severe COVID-19.

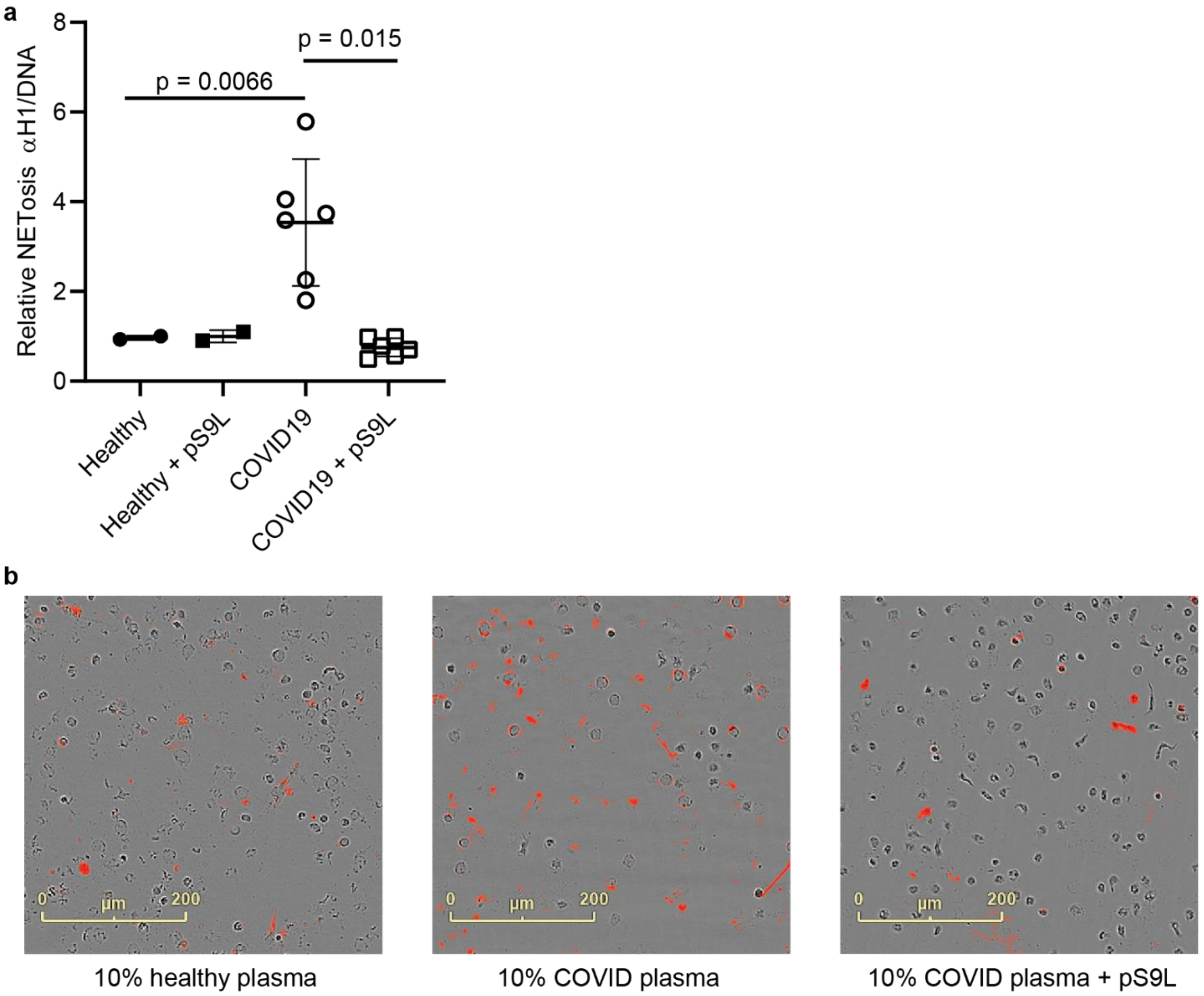

Figure S12. A Siglec-9 agonist inhibits NETosis of neutrophils induced by 10% COVID-19 plasma diluted in media. Neutrophils were isolated from healthy donors and cultured with pS9L (500 nM) in undiluted plasma for 4 h. Plasma was citrate anti-coagulated and from healthy donors or COVID-19 patients and diluted to 10% in IMDM. Cells were then fixed with 4% paraformaldehyde and blocked in 10% goat serum without permeabilization. (**a**) NETs were detected by immunocytochemistry with a mouse anti-H1/DNA complex primary antibody (MAB3864, 1:100) and a goat anti-mouse AlexaFluor594 (1:1000) secondary. Cells were counterstained with MemGlow488 prior to imaging but after immunostaining. The area of all red fluorescent objects >25 µm^2 and >2.0 RCU was quantified and averaged across four images per

well for three wells and normalized to cell count as determined by green fluorescent objects >50 μm^2 and >10.0 GCU. Each data point represents the mean value from technical replicates of an individual donor/patient. Error bars represent SD. Statistics were determined using a mixed effects model to account for differences in neutrophil donors and paired for matched patient plasmas. (**b**) Representative images showing anti-H1/DNA staining.

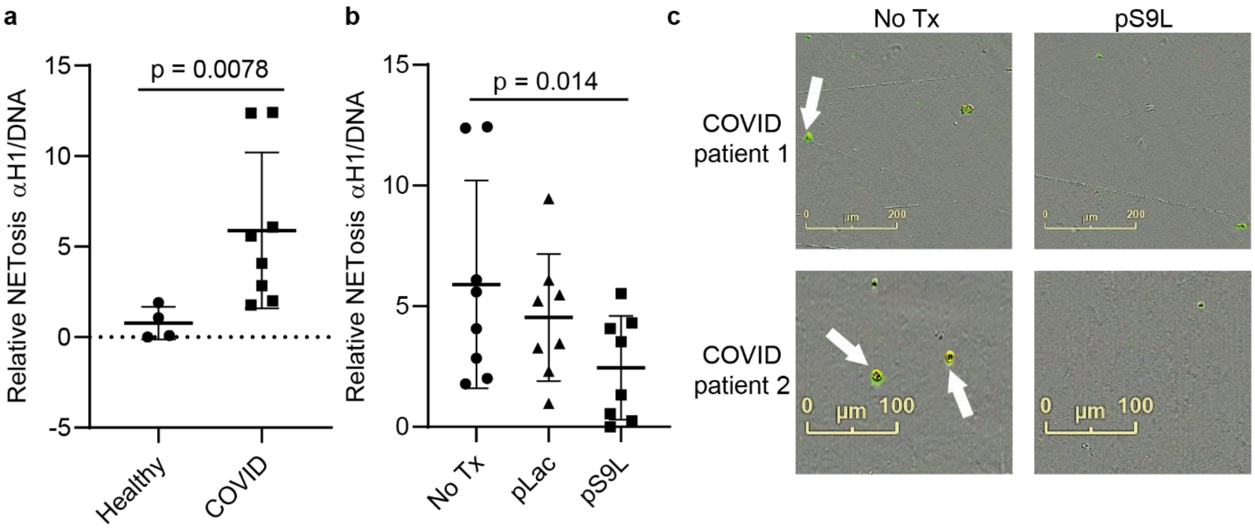

Figure S13. A Siglec-9 agonist inhibits NETosis of neutrophils induced by undiluted COVID-19 plasma. Neutrophils were isolated from healthy donors and cultured with pS9L (500 nM) or pLac (500 nM) in undiluted plasma for 4 h. Plasma was citrate anti-coagulated and from healthy donors or COVID-19 patients. Cells were then fixed with 4% paraformaldehyde and blocked in 10% goat serum without permeabilization. (**a**) NETs were detected by immunocytochemistry with a mouse anti-H1/DNA complex primary antibody (MAB3864, 1:100) and a goat anti-mouse AlexaFluor594 (1:1000) secondary. Cells were counterstained with MemGlow488 prior to imaging but after immunostaining. The area of all red fluorescent objects >25 μm^2 and >2.0 RCU was quantified and averaged across four images per well for three wells and normalized to cell count as determined by green fluorescent objects >50 μm^2 and >10.0 GCU. Each data point represents the mean value from technical replicates of an individual donor/patient. Error bars represent SD. Statistics were determined using a mixed effects model to account for differences in neutrophil donors and paired for matched patient plasmas. (**b**) Representative images showing anti-H1/DNA staining (red) overlayed with MemGlow488 (green).

Extended Materials and Methods.

Safety statement.

For experiments using plasma from patients with COVID-19, all experiments were performed in a certified BSL-2+ biosafety cabinet with appropriate institutional approval for working with blood products derived from patients with COVID-19. All items that came in contact with plasma were disinfected with 10% bleach for 30 min or fixed in 4% formaldehyde solution for 15 min before being removed from the biosafety cabinet. Otherwise, no unexpected or unusually high safety hazards were encountered.

Glycopolypeptide synthesis.

Glycopolypeptides were synthesized as previously described.[41,50] In brief, *N*-carboxyanhydride monomers were polymerized either with a functionalized initiator that affords a membrane-tethering moiety or with a Ni(0) initiator that affords a soluble polypeptide. Polypeptides were deprotected with hydrazine monohydrate and purified by dialysis. In some cases, lactose-bearing scaffolds were then chemoenzymatically functionalized to afford terminal Siglec-9 ligands.[41] All glycopolypeptides were endotoxin purified on Pierce endotoxin removal resin (ThermoFisher, 88274) and sterile filtered (0.22 µm) before use.

Primary cell isolation.

Whole blood samples were obtained from the healthy donors and anti-coagulated with K2EDTA at the Stanford Blood Center. Samples were de-identified by the Stanford Blood Center. Neutrophils were purified on the same day as isolation, and the whole blood samples were kept at room temperature between collection and neutrophil isolation. Neutrophils were isolated by

EasySep Direct Neutrophil Isolation Kit (StemCell Technologies, 19666) according to the manufacturer's instructions in 5 mL aliquots on a magnetized rack (StemCell Technologies, 18103). Efficiency of isolation was determined by flow cytometry to identify CD45 and CD14 hi cells. Neutrophils were routinely obtained in >96% purity via this method.

COVID-19 patients and specimen collection.

Peripheral blood was collected from patients enrolled in the IRB-approved Stanford University Emergency Department COVID-19 Biobank beginning in April 2020 after written informed consent from patients or their surrogates. Eligibility criteria included age ≥18 years and presentation to the Stanford Hospital with a positive SARS-CoV-2 nasopharyngeal swab by RT-PCR. Patients were phenotyped for COVID-19 severity according to the universal World Health Organization (WHO) ordinal scale. Blood draws from patients occurred upon presentation to the Stanford Hospital Emergency Department in concert with usual care to avoid unnecessary personal protective equipment usage. Blood was collected into CPT vacutainers (Becton, Dickinson, and Co.) and plasma isolated, aliquoted, and stored at -80°C after centrifugation at 1800 x g for 20 minutes at 25°C. All sample processing occurred under BSL2+ biosafety precautions as approved by Stanford University APB.

Cell culture.

The acute promyelocytic leukemia cell line HL-60 (ATCC, CCL-240) were cultured in RPMI-1640 supplemented with 20% hiFBS (ThermoFisher, 10-438-026). Cultures were thoroughly washed with prewarmed complete media when bringing out of cryostorage to completely remove DMSO, as residual DMSO can induce differentiation in HL-60s.[66] Cells were allowed to recover for at least two weeks prior to any NETosis experiments. Cultures were not allowed to

exceed a density of 5e5 cells per mL of culture media, as high densities can also induce differentiation.[66]

To induce differentiation of HL-60's, 2e6 cells were harvested by centrifugation (300 rcf, 5 min) and resuspended in complete media (RPMI-1640 +20% hiFBS) supplemented with 100 nM ATRA (Sigma-Aldrich, R2625) and DMSO (Sigma-Aldrich, D8414). Cells were cultured for 5-6 days before use, with a complete media change 48 h prior to use.

NETosis assays.

For live cell NETosis assays were performed in 96 well plates (Corning, 3595) and monitored by fluorescence microscopy with an Incucyte S3 (Essen Biosciences) or an Incucyte ZOOM (Essen Biosciences). DNA staining was performed using membrane impermeable fluorogenic DNA intercalators Cytotox Green (Essen Biosciences, 4633) or Cytotox Red (Essen Biosciences, 4632).

Plates were prepared containing 10X solutions of compounds (20 µL) and a 2X solution of DNA dye (100 µL) in serum-free media (IMDM for primary neutrophils, RPMI-1640 for HL-60s) lacking phenol red. Freshly harvested neutrophils in media (IMDM with 1.2% hiFBS for primary neutrophils for 0.5% final concentration of hiFBS, serum free RPMI-1640 for HL-60s) were then added to the plate (80 µL, 2.5e5 per mL, 2e4 per well) from a cell suspension. The plates were briefly centrifuged at 300 rcf for 1 min to settle the cell suspension, and the plates were immediately transferred to the Incucyte for periodic monitoring (every 10 min for 8 h or every 15 min for 12 h).

For NETosis assays with diluted COVID-19 plasma, freshly isolate neutrophils were plated on 96 well plates (Corning, 3595) coated with 0.01% poly-L-lysine (Sigma, P4707) in IMDM supplemented with 0.5% hiFBS. The neutrophils were allowed to settle and adhere for 20 min before the media was removed and IMDM containing 10% healthy or COVID-19 plasma and with or without glycopolypeptide (500 nM) was added. Alternatively, control wells were treated with no inducer, PMA (500 nM) or R848 (10 µM) in IMDM with 0.5% hiFBS. Plates were incubated for 4 h at 37 °C and the media was gently removed. The cells were then fixed for 15 min at room temperature in a solution of 4% PFA in PBS (ThermoFisher, FB002). The fixed cells were then blocked in 10% goat serum in PBS, stained with anti-H1/DNA (EMD Millipore, MAB3864), and visualized with anti-mouse AlexaFluor 594 (Jackson ImmunoResearch, 115-585-174). Plates were imaged using an Incucyte S3.

For NETosis assays with undiluted COVID-19 plasma, freshly isolate neutrophils were plated on black-walled half-area 96 well plates (Greiner, 675090) coated with 0.01% poly-L-lysine (Sigma, P4707) in IMDM supplemented with 0.5% hiFBS. The neutrophils were allowed to settle and adhere for 20 min before the media was removed and either healthy or COVID-19 plasma and with or without glycopolypeptide (500 nM) was added. Alternatively, control wells were treated with no inducer or R848 (10 µM) in IMDM with 0.5% hiFBS. Plates were incubated for 4 h at 37 °C and the media was gently removed. The cells were then fixed for 15 min at room temperature in a solution of 4% PFA in PBS (ThermoFisher, FB002). The fixed cells were washed. Then cells were then blocked in 10% goat serum. For plates imaged by Incucyte S3, cells were stained with anti-H1/DNA (EMD Millipore, MAB3864) and visualized with a combination of anti-mouse AlexaFluor 594 (Jackson ImmunoResearch, 115-585-174) and MemGlow488 (Cytoskeleton, MG01-02). For plates imaged by immunofluorescence using a

Keyence BZ-710, cells were stained with anti-MPO (Thermo Scientific, MA516383) and visualized with anti-rabbit AlexaFluor 555 (Thermo Scientific, A27039). Cells were imaged in HBSS containing DAPI imaging solution (Thermo Scientific, R37606). Images were collected using a Keyence BZ-X710 and images were analyzed using ImageJ.

Incucyte images were analyzed using the onboard Incucyte analysis software. For Incucyte S3 analyses: phase images were analyzed to identify cells using the following settings: segmentation adjustment = 0.8; minimum area filter = 25 μm^2. For primary neutrophils, fluorescence areas were determined using the following settings: top hat segmentation; radius (µm) = 10.0; threshold = 10.0 GCU or 0.1 RCU; area filter = 300 μm^2. For dHL-60s, fluorescence areas were determined as for primary neutrophils with the following variation: area filter = 200 μm^2. For Incucyte ZOOM analyses: phase images were analyzed to identify cells using the following settings: segmentation adjustment = 0.8; minimum area filter = 25 μm^2. For primary neutrophils, fluorescence areas were determined using the following settings: top hat segmentation; radius (µm) = 10.0; threshold = 5.0 GCU; area filter = 200 μm^2

Reactive oxygen species measurements.

Induction of reactive oxygen species was measured by fluorescence microscopy using an Incucyte S3 (Essen Biosciences). Cells were prepared as for NETosis assays, with the difference that rather than the inclusion of Cytotox dye in the media, CellROX Deep Red (Thermo Fisher, C10422) was included at a final concentration of 5 µM. Data were analyzed as in NETosis assays, with the following changes in settings for quantifying red fluorescence: top hat segmentation; radius (µm) = 10.0; threshold = 1.6 RCU; area filter = 25 μm^2.

siRNA knock downs.

For siRNA knock down experiments, HL-60's were differentiated as indicated above. On day 4, the media was changed and 3 mL of culture was transfected with 30 pmol siRNA's (IDT) using Lipofectamine RNAiMAX (Thermo Fisher, 13778100) according to the manufacturer's instructions. Transfectants were cultured for 48 h prior to use in NETosis assays, with a complete media change at 24 h post transfection. Protein expression was validated by Western blot or flow cytometry, as applicable.

For siRNA KD of SIGLEC9, the top two suggested predesigned dicer substrate siRNA's from IDT (hs.Ri.SIGLEC9.13.1 and hs.Ri.SIGLEC9.13.2) were combined in a 1:1 molar ratio. For siRNA KD of PTPN6, the top suggested predesigned dicer substrate siRNA (hs.Ri.PTPN6.13.1) was used. As a negative control, scramble siRNA's were obtained from IDT.

Flow cytometry.

All flow cytometry experiments were performed using a BD Accuri 6 flow cytometer and analyzed using FlowJo 10. For all flow cytometry experiments, live cells were isolated from culture and resuspended in cold PBS supplemented with 1% BSA at a concentration of 1e7 cells per mL. Aliquots of 5e5 cells were then stained at the concentrations indicated in the Reagents Table and Usage for 30 min on ice in the dark. Cells were then washed three times (500 rcf, 5 min) with in cold PBS supplemented with 1% BSA before being analyzed by flow cytometry.

SDS-PAGE and Western blotting.

Cell pellets were washed twice with cold PBS supplemented with 1 mM EDTA and were lysed in RIPA buffer (50 µL for each 1e6 cells) (Thermo Fisher, PI89900) supplemented with Benzonase (1:1000) (Sigma Aldrich, E1014), HALT phosphatase inhibitor (1:100) (Thermo Fisher, 78420), and HALT protease inhibitor (1:100) (Thermo Fisher, 87786) for 30 min at 4 °C. Lysates were clarified by centrifugation at 4 °C for 15 min at 16000 rcf. Protein concentration was quantified by Rapid Gold BCA (Thermo Fisher, A53226).

Lysates were diluted into SDS-buffer with beta-mercaptoethanol and were separated by SDS-PAGE on 4-12% bisacrylamide gels loading 10 µg per lane. For analysis of histone H3 citrullination, lysates were separated in XT-MES at 200 V for 35 min. For analysis of SHP-1 levels after siRNA KD, lysates were separated in XT-MOPS at 200 V for 1 h. Blots were transferred to nitrocellulose and blocked with 5% BSA in TBS before being stained with primary antibodies and IR-dye conjugated secondary antibodies for analysis by LiCOR.

Protein expression and purification.

All proteins for enzymatic synthesis of glycopolypeptides were expressed and purified as previously described.[41]

His-tagged proteins cultures were grown at 37 °C to an OD600 of 0.8-1.0 in 1 L of LB containing the appropriate antibiotic selection marker, at which point expression was induced with IPTG (0.1 mM) and cultures were grown overnight at 20 °C with shaking at 220 rpm. After 24h, cells were pelleted by centrifugation and lysed in buffer (250 mM TrisHCl, 0.5 M NaCl, 20 mM imidazole, 0.1% TritonX100) supplemented with protease inhibitor cocktail (Sigma Aldrich, 04693132001) (one tablet per 40 mL) and DNAseI (Thermo Fisher, 90083) (10 µL per 40 mL). Cells were lysed using a dounce homogenizer followed by French press. Lysates were clarified

by centrifugation and purified on HisTRAP columns (GE Life Sciences, 17-5247-01) using a gradient of 20 mM to 200 mM imidazole on an AKTA FPLC. Fraction purity was determined by SDS-PAGE and pure fractions were combined, purified by dialysis against storage buffer (50 mM Tris HCl, 250 mM NaCl, 10% glycerol), aliquoted, and flash-frozen in liquid nitrogen for storage in a -80 °C freezer.

Phosphoproteomics.

Primary neutrophils were treated in media containing the indicated compounds in IMDM [+] 0.5% hiFBS for the indicated time period, or in plasma from either healthy donors or COVID-19 patients, with or without pS9L (500 nM) for 15 min. Cell pellets were washed twice with cold PBS supplemented with 1 mM EDTA and were lysed in RIPA buffer (50 uL for each 1e6 cells) (Thermo Fisher, PI89900) supplemented with Benzonase (1:1000) (Sigma Aldrich, E1014), HALT phosphatase inhibitor (1:100) (Thermo Fisher, 78420), and HALT protease inhibitor (1:100) (Thermo Fisher, 87786) for 30 min at 4 °C. Lysates were clarified by centrifugation at 4 °C for 15 min at 16000 rcf. Protein concentration was quantified by Rapid Gold BCA (Thermo Fisher, A53226).

Digestion was performed on 100 µg protein using a mini S-trap protocol provided by the manufacturer (Protifi).[67] Here, proteins brought to 5% SDS and reduced with 5 mM DTT for 10 minutes at 95 C. Cysteines were alkylated using 30 mM iodoacetamide for 45 minutes each at room temperature in the dark. The lysate was then acidified with phosphoric acid, brought to approximately 80-90% methanol with 100 mM TEAB in 90% methanol, and loaded onto the S-trap column. Following washing with 100 mM TEAB in 90% methanol, trypsin (Promega) was added to the S-trap at a 20:1 protein:protease ratio for 90 minutes at 47 °C. Peptides from each lysate were labeled with 11-plex TMT (Tandem Mass Tags, Thermo Fisher Scientific) for 2

hours at room temperature using recently published protocols.[47,68] Labeling schemes for the stimulated study comparing R848 and PMA to no treatment (NT) were: NT replicates in channels 126C (Donor 1), 127N (Donor 2), and 130C (Donor 3); PMA replicates in 127C (Donor 1), 128N (Donor 2) and 131N (Donor 3); R848 replicates in 128C (Donor 1), 129N (Donor 2) and 131C (Donor 3). For the polymer experiment, the labeling scheme was: vehicle replicates in channels 126C (Donor 1), 127N (Donor 2), and 130C (Donor 3); pS9L replicates in 127C (Donor 1), 128N (Donor 2) and 131N (Donor 3); pLac replicates in 128C (Donor 1), 129N (Donor 2) and 131C (Donor 3). A test mix was run to confirm >99% labeling efficiency and even distribution of signal across all channels prior to quenching of the TMT labeling reaction (0.5 uL 50% hydroxylamine reacted for 15 min). Peptides from each channel were then combined prior to phosphopeptide enrichment, which was performed as previously described.[69] Briefly, 100 µL magnetic titanium(IV) immobilized metal ion affinity chromatography (Ti(IV)-IMAC, ReSyn Biosciences) beads were washed three times with 1 mL 80% acetonitrile/6% TFA (all washes were 1 mL).[70] Peptides were dissolved in 1 mL 80% acetonitrile/6% TFA and gently vortexed with the TI(IV)-IMAC beads for 45 minutes. Unbound peptides were kept as flow through for total protein analysis, followed by three 80% acetonitrile/6% TFA, one 80% acetonitrile, one 0.5 M glycolic acid/80% acetonitrile, and two 80% acetonitrile washes. Peptides were eluted with 500 µL 50% acetonitrile, 1% ammonium hydroxide. Both eluate and flow through were dried down in a speed vac and further cleaned up on Strata-X SPE cartridges (Phenomenex) by conditioning the cartridge with 1 mL ACN followed by 1 mL 0.2% formic acid (FA) in water. Peptides were resuspended in 0.2% FA in water and then loaded on to the cartridge, followed by a 1 mL wash with 0.2% FA in water. Peptides were eluted with 400 uL of 0.2% FA in 80% ACN, were dried via lyophilization.

All samples were resuspended in 0.2% formic acid in water prior to LC-MS/MS analysis. Total protein samples were resuspended in 500 µL with 1 µL injected on column, while enriched phosphopeptides were resuspended in 15 µL total with 4 µL injected per analysis. Triplicate injections were collected for all samples. All (phospho)peptide mixtures were separated over a 25 cm EasySpray reversed phase LC column (75 µm inner diameter packed with 2 µm, 100 Å, PepMap C18 particles, Thermo Fisher Scientific). The mobile phases (A: water with 0.2% formic acid and B: acetonitrile with 0.2% formic acid) were driven and controlled by a Dionex Ultimate 3000 RPLC nano system (Thermo Fisher Scientific). An integrated loading pump was used to load peptides onto a trap column (Acclaim PepMap 100 C18, 5 um particles, 20 mm length, Thermo Fisher Scientific) at 8 µL/min, which was put in line with the analytical column 4 minutes into the gradient for the total protein samples. The gradient increased from 0% to 5% B over the first 4 minutes of the analysis, followed by an increase from 5% to 25% B from 4 to 158 minutes, an increase from 25% to 90% B from 158 to 162 minutes, isocratic flow at 90% B from 162 to 168 minutes, and a re-equilibration at 0% for 12 minutes for a total analysis time of 180 minutes. Eluted (phoshpo)peptides were analyzed on an Orbitrap Fusion Tribrid MS system (Thermo Fisher Scientific). Precursors were ionized using an EASY-Spray ionization source (Thermo Fisher Scientific) source held at +2.2 kV compared to ground, and the column was held at 40 °C. The inlet capillary temperature was held at 275 °C. Survey scans of peptide precursors were collected in the Orbitrap from 350-1350 Th with an AGC target of 1,000,000, a maximum injection time of 50 ms, and a resolution of 60,000 at 200 m/z. Monoisotopic precursor selection was enabled for peptide isotopic distributions, precursors of z = 2-5 were selected for data-dependent MS/MS scans for 2 second of cycle time, and dynamic exclusion was set to 30 seconds with a ±10 ppm window set around the precursor monoisotope. An isolation window of 1 Th was used to select precursor ions with the quadrupole. MS/MS scans were collected using HCD at 30 normalized collision energy (nce) with an AGC target of 100,000 and a maximum

injection time of 118 ms. Mass analysis was performed in the Orbitrap with a resolution of 60,000 with a first mass set at 100 Th.

Phosphoproteomic data analysis.

All data were searched with the Andromeda search engine[71] in MaxQuant[72] using the entire human proteome downloaded from Uniprot[73] (reviewed, 20428 entries). Each separate TMT experiment (resting, activated, and pLac control) was searched separately, with the flow through/total protein triplicate injections labeled as Group0 and False under “PTM” and phosphopeptide enriched triplicate injections labeled as Group1 and True under “PTM”. Group0 had cleavage specificity set to Trypsin/P with 2 missed cleavage allowed and variable modifications of oxidation of methionine and acetylation of the protein N-terminus with 4 maximum modifications per peptide. Group1 had cleavage specificity set to Trypsin/P with 3 missed cleavage allowed and variable modifications of phosphorylation on serine/threonine/tyrosine, oxidation of methionine, and acetylation of the protein N-terminus with 4 maximum modifications per peptide. The experiment type for both Group0 and Group1 was set to Reporter ion MS2 and only TMT channels used (as described above) were selected to be included. The reporter ion mass tolerance was set to 0.3 Da and the minimum reporter PIF score was set to 0.75. Defaults were used for the remaining settings, including PSM and protein FDR thresholds of 0.01 and 20 ppm, 4.5 ppm, and 20 ppm for first search MS1 tolerance, main search MS1 tolerance, and MS2 product ion tolerance, respectively. Match between runs was not enabled. Quantified phosphosites were then processed in Perseus.[74] Contaminants and reverse hits were removed, results were filtered for phosphosites that had localization probabilities > 0.75, and signal in all relevant TMT channels was required. Significance testing was performed using a two-tailed pair-ed sample t-test calculated in Microsoft Excel, using one condition versus control (NT or vehicle for stimulated and polymer experiments, respectively) for

pairwise comparisons. Data have been deposited to the ProteomeXchange Consortium via the PRIDE partner repository with the dataset identifier PXD022990.[75]

Analysis of publicly-available single-cell RNA-sequencing (scRNA-seq) data.

The open source statistical software R (www.r-project.org; v3.6.1) and the R package Seurat (v 3.2.2) was used for scRNA-seq data analysis.[76] A pre-processed Seurat object containing scRNA-seq data and metadata of neutrophils profiled using the BD Rhapsody platform was retrieved from www.fastgenomics.org as outlined in the data availability statement of Schulte-Schrepping, et al.[8] The average expression of *SIGLEC9* or *PADI4* was defined as the mean of log-normalized transcript counts, calculated by NormalizeData() function, in a given sample. DotPlot() was used to visualize average and percent expression of *SIGLEC9* or *PADI4*.

Reagent Table and Usage.

IN – Incucyte S3 (microscopy); OC – Octet (*in vitro* protein binding); FC – flow cytometry; WB – western blot; KD – siRNA knock down; IF – immunofluorescence

**Reagent**	**Source (#)**	**Usage, dilution/concentration**
Cytotox Green	Essen Biosciences (4633)	IN, 1:4,000
Cytotox Red	Essen Biosciences (4632)	IN, 1:4,000
CellROX Deep Red	ThermoFisher (C10422)	IN, 1:500
Anti-Siglec-9 clone K8 / AlexaFluor 647	BioLegend (351509)	FC, 1:50
Anti-human CD45 clone HI30 / APC	Stemcell Tecnologies (60018AZ.1)	FC, 1:50
Anti-human CD16 clone 3G8 / AlexaFluor 488	Stemcell Technologies (60041AD.1)	FC, 1:50

Mouse IgG1 isotype clone MOPC-21 / FITC	BD Biosciences (551954)	FC, 1:50
Mouse IgG1 isotype clone MOPC-21 / APC	BD Biosciences (550854)	FC, 1:50
Siglec-9-Fc	R&D Systems (1139-SL-050)	OC, 400 nM
DsiRNA (SIGLEC9)	IDT (hs.Ri.SIGLEC9.13.1)	KD, 30 nM
DsiRNA (SIGLEC9)	IDT (hs.Ri.SIGLEC9.13.2)	KD, 30 nM
DsiRNA (PTPN6)	IDT (hs.Ri.PTPN6.13.1)	KD, 30 nM
siRNA negative control	IDT (51-01-19-08)	KD, 30 nM
Rabbit anti-CitH3 (R2/R8/R17)	Abcam (ab5103)	WB, 1:1,000
Mouse anti-GAPDH	Sigma-Aldrich (G8795-100UL)	WB, 1:10,000
NSC-87877	Sigma-Aldrich (565851-50MG)	IN, 50 µM
Rabbit anti-SHP1 (clone Y476)	Abcam (ab32559)	WB, 1:1,000
goat anti-mouse 680RD	LiCOR (926-68070)	WB, 1:10,000
goat anti-rabbit 800CW	LiCOR (926-32211)	WB, 1:10,000
anti-H1/DNA	EMD Millipore (MAB3864)	IF, 1:100
goat anti-mouse AlexaFluor 594	Jackson ImmunoResearch (115-585-174)	IF, 1:1000
MemGlow488	Cytoskeleton (MG01-02)	IF, 1:200
anti-myeloperoxidase clone SP72	Thermo Scientific (MA516383)	IF, 1:100
goat anti-rabbit AlexaFluor 555	Thermo Scientific (A27039)	IF, 1:1000
DAPI solution	Thermo Scientific (R37606)	IF, 2 drops per mL
